# Supplementary material for: Seamless optical cloud computing across edge-metro network for generative AI
Source: Nat Commun. 2025 Jul 2;16:6097. doi: 10.1038/s41467-025-61495-6 (PMC12223312; doi:10.1038/s41467-025-61495-6)
Supplement: Supplementary file 1 — Supplementary Information [file 41467_2025_61495_MOESM1_ESM.pdf]

Supplementary Information for

Seamless Optical Cloud Computing across Edge-Metro Network for Generative AI

Sizhe Xing[1,2,3,†], Aolong Sun[1,3,†], Chengxi Wang[1,3,†], Yizhi Wang[2], Boyu Dong[1,3], Junhui Hu[1,3], Xuyu Deng[1,3], An Yan[1,3], Yinjun Liu[1,3], Fangchen Hu[4], Zhongya Li[1,3], Ouhan Huang[1,3], Junhao Zhao[1,3], Yingjun Zhou[1,3], Ziwei Li[1,3], Jianyang Shi[1,3], Xi Xiao[5], Richard Penty[2], Qixiang Cheng[2,*], Nan Chi[1,3,*], Junwen Zhang[1,3,*]

[1]School of Information Science and Technology, Fudan University, Shanghai, China

[2]Centre for Photonic Systems, Electrical Engineering Division, Department of Engineering, University of Cambridge, Cambridge CB3 0FA, UK.

[3]Key Laboratory for Information Science of Electromagnetic Waves (MoE), Fudan University, Shanghai, China

[4]Zhangjiang Laboratory, Shanghai, China

[5]National Information Optoelectronics Innovation Center, Wuhan 430074, China

[†]*These authors contributed equally to this work.*

**Corresponding authors*: junwenzhang@fudan.edu.cn, qc223@cam.ac.uk, nanchi@fudan.edu.cn

Contents

Supplementary Note 1: The design method of the AWGR-based processing unit 3

Supplementary Note 2: The test and calibration of the AWGR 5

Supplementary Note 3: Principles of AWGR-based Convolution Computation 5

Supplementary Note 4: Image Dimensionality Reduction Strategy 7

Supplementary Note 5: Principle of Loading Both Positive and Negative Weight Based on Microring-Enhanced AWGR 10

Supplementary Note 6: OPU Convolution Accuracy Measurement............................ 11

Supplementary Note 7: The Experiment of Optical Communication.......................... 12

Supplementary Note 8: Details and Results of Optical Cloud Computing.................. 13

Supplementary Note 1: The design method of the AWGR-based processing unit

Fig. S1 shows the structural composition of the AWGR, consisting of two star couplers and an array of waveguides. Both star couplers have identical configurations, where the input ports of star coupler A also serve as the output ports of star coupler B. Each star coupler is formed by two intersecting circles, where the radius of the smaller circle is half that of the larger circle. Fig. S1b illustrates the design schematic of the input star coupler. When light enters through port p and exits through port q, the optical path difference encountered by the light at adjacent channels in the rectangular waveguide at the input port p and the optical path difference at the output port q are as follows:

$$\Delta l_{i\_p} = n_s l_{m\_p} - n_s l_{m-1_p} = n_s d_a \sin\theta_{\mathrm{i\_p}} = \frac{n_s p d_i d_a}{R} \quad (1)$$

$$\Delta l_{o\_q} = n_s l_{m\_q} - n_s l_{m-1_q} = n_s d_b \sin\theta_{\mathrm{o\_q}} = \frac{n_s q d_o d_b}{R} \quad (2)$$

The terms d_i, d_a, d_b *and* d_o represent the distances between the input port of input star coupler A, the output port of star coupler A, the input port of star coupler B, and the output port of star coupler B, respectively. L_{m_p} denotes the path length from the input port p to the output port m of the star coupler. N_s is the refractive index of the star coupler, and R is the radius of the larger circle, which is also the diameter of the small circle. Consequently, the optical path difference between the two adjacent paths from input port p to output port q is as follows:

$$\Delta l_{p\_q} = \Delta l_{i\_p} - \Delta l_{i_q} + n_c \Delta l_m = \frac{n_s p d_i d_a}{R} - \frac{n_s q d_o d_b}{R} + n_c \Delta l_m \quad (3)$$

Where the terms Δl_m and n_c represent the length intervals and the refractive index of the waveguide array, respectively. Assuming that constructive interference occurs when light of wavelength λ_0 travels from input port 0 to output port 0, the following condition must be satisfied:

$$\Delta l_{0\_0} = n_c(\lambda_0)\Delta l_m = k\lambda_0 \quad (4)$$

Assuming that the wavelength $\lambda_{p,q}$ allows light to enter through port p and exit through port q, it must satisfy the following condition:

$$k\lambda_{p,q} = \frac{n_s(\lambda_{p,q}) p d_i d_a}{R} - \frac{n_s q d_o d_b}{R} + k\lambda_0 \frac{n_c(\lambda_{p,q})}{n_c(\lambda_0)} \quad (5)$$

Therefore, it can be determined that the wavelengths of light entering adjacent ports p and p-1 and exiting from port q follows:

$$\lambda_{p,q} - \lambda_{p\text{-}1,\,q} = \lambda_0 \frac{n_c(\lambda_{p,q}) - n_c(\lambda_{p-1,q})}{n_c(\lambda_0)} + \frac{\left(n_s(\lambda_{p,q}) - n_s(\lambda_{p-1,q})\right)(p d_i d_a - q d_o d_b)}{kR} + \frac{n_s(\lambda_{p-1,q}) d_i d_a}{kR} \quad (6)$$

Since $\lambda(p,q)$ and $\lambda(p,q)$ are closely adjacent, it can be approximated that:

$$\frac{n_c(\lambda_{p,q}) - n_c(\lambda_{p-1,q})}{\lambda_{p,q} - \lambda_{p\text{-}1,\,q}} = \frac{dn_c}{d\lambda} \quad (7)$$

$$\frac{n_s(\lambda_{p,q}) - n_s(\lambda_{p-1,q})}{\lambda_{p,q} - \lambda_{p\text{-}1,\,q}} = \frac{dn_s}{d\lambda} \quad (8)$$

Given that the group dispersion n_g is defined as $n_g = n_c(\lambda_0) - \lambda_0 \frac{dn_c(\lambda)}{d\lambda}$, it follows that:

$$n_g = \frac{n_s(\lambda_{p-1,q})n_c(\lambda_0)d_i d_a}{kR(\lambda_{p,q}-\lambda_{p\text{-}1,\,q})} + \frac{(pd_i d_a - qd_o d_b)}{kR}\frac{dn_s(\lambda)}{d\lambda} \tag{9}$$

By omitting the smallest term in the series, the interval between the wavelengths can be calculated as:

$$\lambda_{p,q} - \lambda_{p\text{-}1,\,q} = \frac{n_s(\lambda_{p-1,q})n_c(\lambda_0)d_i d_a}{kRn_g} \tag{10}$$

Similarly, it can be derived:

$$\lambda_{p,q} - \lambda_{p,q-1} = -\frac{n_s(\lambda_{p,q\text{-}1})n_c(\lambda_0)d_o d_b}{kRn_g} \tag{11}$$

For an AWGR, there exists a scenario where two adjacent orders of wavelengths share the same propagation path. The interval between these two wavelengths is defined as the free spectral range (FSR),

$$\Delta FSR = \frac{n_c\lambda}{kn_g} \tag{12}$$

To achieve the objective of wavelength routing, the number of channels within one FSR should satisfy the following condition:

$$N_{ch} = \frac{\Delta FSR}{\Delta\lambda} = \frac{n_c\lambda}{\Delta\lambda k n_g} \tag{13}$$

Scalability of the AWGR-based processing unit

The approach of using an AWGR for convolution computations has a limitation in that the size of the device is large and, theoretically, difficult to significantly reduce. By combining Equations (10) and (12), it can be determined that:

$$R = \frac{n_s d_i d_a \Delta\lambda_{FSR}}{\Delta\lambda\lambda_0} \tag{14}$$

This indicates that increasing the number of channels will significantly enlarge the device size due to the expanded FSR. The specific impacts are illustrated in Fig. S2. Fig. S2a shows the AWGRs with a 50 GHz channel spacing at varying channel numbers, while the Fig. S2b displays the corresponding dimensions for a 100 GHz channel spacing. From the figure, it can be observed that as the channel number increases, the device size grows accordingly. This scaling is primarily driven by the elongation of the short side caused by the expanded star coupler structure. Notably, even in a 64×64 configuration, the short side length remains below 1.8 cm. Table S1 quantifies the impact of channel number scaling on device size and peak computational capacity under a fixed channel spacing of 100 GHz. As the number of channels increases from 4 to 64, the device size scales by 400%, while the computational speed exhibits a remarkable 25,500% enhancement. This results in a significant improvement in computational efficiency per unit area. Although enlarging the AWGR introduces a moderate increase in physical footprint, the substantial gains in computational throughput justify this trade-off, demonstrating the scalability and practical viability of the proposed architecture.

To ensure that all emitted light is coupled into the waveguide array, the divergence angle of the incident light must be considered. Consequently, the required number of arrayed waveguides is:

$$N = N_A\frac{R}{d_a} = N_A\frac{n_s d_i \Delta\lambda_{FSR}}{\Delta\lambda\lambda_0} \tag{15}$$

Where N_AR represents the length over which the incident light scatters across the output face of the star coupler. Under the same design method, the volume of the rectangular waveguide depends on the product of the number of waveguides and the difference in length between them:

$$N\Delta l_m = N_A \frac{n_s d_i \lambda_0}{\Delta\lambda n_g} \tag{16}$$

Under the same medium and wavelength band, $n_s, \lambda_0, and\ n_g$, remain constant, while the minimum value of d_i depends on the mode field size in the transmission medium. Therefore, the size of the rectangular waveguide is always the same for the same wavelength interval. To significantly reduce the size of the AWGR in the future, options would include using a larger channel spacing or adopting an entirely new design approach.

Supplementary Note 2: The test and calibration of the AWGR

Fig. S2 displays the transmission spectra for each input and output port of an 8x8 AWGR. Each color in the figure represents the transmission spectrum of one output channel. The channel spacing shown is 84 GHz, with a FSR of 672 GHz, which is eight times the channel spacing. It is also observed that the spectral interval for light emitted from the same output channel corresponds exactly to one channel spacing for adjacent input channels. Additionally, the transmission spectra exhibit jitter as the wavelength changes, which is attributed to the insufficient divergence coefficient selected in Equation 15. This results in more light being coupled into the waveguide array from the 0th level port than from the *p* ports.

In the experiments, to reduce the size of the AWGR, The light scatter factor of 1.6 was used, which might not be sufficient for complete coupling of all light. To mitigate the impact of this on the experiment, Table S2 documents the received optical power of a broadband light source transmitted between every two ports and uses this data to calibrate the input light for each port. From the table, it is evident that the data generally exhibits a piled distribution, with symmetric ports displaying similar transmission losses.

In the experiment, a broadband light source with a power of 8 dBm was emitted. Due to the filtering effect of the AWGR and losses caused by the grating couplers, the output optical power was only about -30 dBm. The minimum optical power was -30.6 dBm and the maximum was -28.7 dBm, where a difference of 1.9 dB is observed. The two ports at the extreme edges experienced significantly higher losses than the others. Fortunately, data is typically not output from these two ports in the experiments. By adjusting the input optical power for each port, this imbalance was addressed in the experiments.

Supplementary Note 3: Principles of AWGR-based Convolution Computation

The wavelength routing relationship of the AWGR can be summarized as illustrated in Fig. S3, where λ represents each transmission peak. The wavelength from port 1 input and port 8 output is defined as λ_1, and the remaining wavelengths output from port 8

are sourced from input ports 2, 3, etc. This provides the wavelength routing relationship depicted in the figure. The routing of three wavelengths is highlighted in the figure. It is evident that these three wavelengths exhibit a continuous shift along the direction of the input ports, which corresponds to the convolution mode. Signals can be loaded along the input ports and weights pre-loaded onto the wavelengths, allowing the convolution results to be sequentially obtained along the output ports. Assuming the signal on each wavelength is denoted as $\sqrt{\omega_i}\exp(j2\pi f_i t)$, and each input port iii maps to the input light as f_i, the signal at each input port follows:

$$I_k(t) = \sum_{i=1}^{m} f_i\left(\sqrt{\omega_i}\exp(j2\pi f_i t)\right),\ m\epsilon\mathbb{Z}^+ \tag{17}$$

Among them, *m* represents the number of wavelengths at the input port. Based on the mapping relationships shown in Equations (10) and (11), the signals at the output ports can be expressed as follows:

$$O_j = \sum_{i=1}^{m} f_{i+j-1}\left(\sqrt{\omega_i}\exp(j2\pi f_i t)\right) \tag{18}$$

Here, O_1 is the output of port 8, and O_2 is the output of port 7, and so on. When $f_i(a,t) = x_i(t) + b$, the output is:

$$O_j = \sum_{i=1}^{m}(x_{i+j-1}(t) + b)\sqrt{\omega_i}\exp(j2\pi f_i t) \tag{19}$$

The signal undergoes square-law detection in the photodetector, and the output signal is:

$$y_j = O_j^2 = \sum_{i=1}^{m}\left(x_{i+j-1}(t) + b\right)^2\omega_i = \sum_{i=1}^{m}(2x_{i+j-1}(t)\cdot b + x_{i+j-1}(t)^2 + b^2)\,\omega_i = \sum_{i=1}^{m} 2bx_{i+j-1}(t)\omega_i \tag{20}$$

In the equation, the squared terms represent the DC component and the squared signal component, respectively. Since the squared signal component is too small to be omitted. Consequently, it can be observed that the output *y* at each port at any given time is the convolution of the input *x* at each port with the weight of ω.

It is worth noting that the figure only shows eight wavelengths within a single cycle. These can also be seen as representatives of wavelengths within each FSR that follow the same transmission pattern. For example, $\lambda_1$and $\lambda_1 + m\lambda_{FSR}, m \in \mathbb{Z}$have the same transmission relationship. Therefore, λ_1 and λ_8 are also adjacent, making the entire wavelength range cyclic.

Fig. S4 illustrates the process of performing convolution operations on a 4x4 AWGR, utilizing three wavelengths and four input ports. The diagram only depicts the principle of optical convolution computation within a single FSR period using the OPU. When a signal of length 4 is convolved with a kernel of length 3, the output signal length is calculated as 4-3+1=2. The computational core in the diagram is a 4×4 AWGR, utilizing three wavelengths for the convolution computation. The signal is loaded through the MZM array on the left, routed through the AWGR for wavelength routing, and finally output through the PD array on the right. Since the length of the output signal is 2, only the top two PDs are actually needed. After removing the constants, the output signal from the structure shown in the diagram is:

$$O = a_1 a_2 (A * \omega) + a_1 b_2 A \tag{21}$$

Where the a_1 and a_2 represent the modulation depth of the signal and the b_1 and b_2 denote to the bias. In the equation, A is a high-speed data, while ω remains constant. Therefore, $a_1 b_2 A$ can distort the received signal. So, it is necessary to set b_2 to zero to eliminate this term. Since the $P = a_2 \cdot \omega + b_2$ is positive item, the ω is also positive when b_2 is equal to 0. Therefore, an unavoidable drawback of the structure depicted in the diagram is that it can only accommodate the loading of positive weights.

Beyond the superior efficiency analyzed in the main manuscript, this architecture offers the additional advantage that, when scaling up to meet the demands of larger computational tasks, the power efficiency can be further improved, as demonstrated in Fig. S5. The computational efficiency of an individual OPU gradually approaches a limit of approximately 15 mW/TOPs, which represents roughly a one-order-of-magnitude improvement compared to the previously reported value of 118.6 mW/TOPs. By selecting an optimal number of wavelengths for each node, the overall computational efficiency can be the best. Consequently, our architecture naturally achieves superior computational efficiency under increased computational demands, highlighting its substantial suitability and adaptability for advanced generative AI workloads.

Supplementary Note 4: Parallelization strategy in cloud optical computing

In this framework, we propose the method to decompose complex computations into multiple simpler and relatively independent subtasks, which can be processed simultaneously. This decomposition includes both the parallel execution of multiple convolutional operations in a large scale model and the dimensional reduction of large-scale convolutions into low-dimensional convolutions processable by individual OPUs. By harnessing the multidimensional parallelism across time, frequency, wavelength, and spatial domains, our architecture achieves hyper-dimensional parallel computation, enabling the handling of all parameters in large-scale models while maintaining high scheduling flexibility.

Within the design, the complete weights of a single convolutional kernel are loaded within one frequency cycle during a single time slot, as shown in Fig. S6. Leveraging wavelength-division multiplexing, multiple weight sets can be simultaneously loaded across distinct spectral channels. By assigning different kernels to separate wavelength ranges, different convolution operations are executed concurrently. In the cloud computing center, optical signals are dynamically routed to dedicated OPUs based on their wavelengths to fulfill parallel computational demands. A distributed array of OPUs is strategically deployed in the cloud optical computing center to support high-speed edge computations. As shown in the figure, results are generated in the computing center and transmitted back to the edge with ultralow latency. This architecture ensures minimal overhead through two key mechanisms: (1) electrical-domain data aggregation under clock synchronization eliminates the need for additional optoelectronic conversions, and (2) task partitioning and OPU coordination avoid redundant data storage. While constrained OPU resources may introduce queuing delays for sequential subtasks, latency remains dominated by computational queuing rather than data I/O

operations. Regarding the implementation of nonlinear layers, the approach most optimally aligned with our architecture leverages the inherent nonlinearity of photodetectors, a strategy has been widely studied in prior works[1,2]. This method enables the integration of nonlinear activation functions without introducing additional device complexity or power overhead. However, given the diversity and precision requirements of nonlinear activation functions (e.g., ReLU, sigmoid), implementing these functions in the electrical domain may serve as a viable alternative. While this introduces a trade-off between photonic integration density and computational flexibility, it ensures compatibility with conventional deep learning frameworks and facilitates precise activation shaping through programmable electronic circuits.

Given that convolutional tasks may exceed the processing capacity of a single OPU, this work further investigates the partitioning of complex convolutions into low-dimensional sub-convolutions for distributed execution across multiple OPUs. This task decomposition strategy enables efficient processing of the image datasets presented in this study, achieving scalable performance while preserving computational accuracy. Since images are two-dimensional data, in this method, they are difficult to directly input into the computational module for processing. However, performing convolution operations on images is a crucial step in the distributed machine learning framework. Therefore, this paper specifically studies and proposes a strategy that can decompose a two-dimensional convolution into several one-dimensional convolutions. This involves the preparation of image data and its adaptation to the specific requirements of the convolution operation within the optical computing framework. The loading process is crucial for ensuring that the spatial features of the image are accurately represented and processed through the system. Assuming the dimensions of the image to be processed are $n_1 \times n_2$, it can be expressed as:

$$\mathbf{A} = \begin{bmatrix} a_{11} & \cdots & a_{1,n_2} \\ \vdots & \ddots & \vdots \\ a_{n_1,1} & \cdots & a_{n_1,n_2} \end{bmatrix} \tag{22}$$

In a convolutional neural network, matrix should be convolved with an m dimensional convolution kernel. The convolution kernel W can be written as:

$$\mathbf{W} = \begin{bmatrix} \omega_{1,1} & \cdots & \omega_{1,m} \\ \vdots & \ddots & \vdots \\ \omega_{m,1} & \cdots & \omega_{m,m} \end{bmatrix} \tag{23}$$

However, the convolution architecture proposed in this paper is not capable of processing two-dimensional convolution operations. Therefore, it is necessary to reduce the dimensionality to one dimension. The paper employs a matrix segmentation algorithm that converts two-dimensional convolution operations into mmm one-dimensional convolutions, where mmm represents the dimension of the convolution kernel. By generating m submatrices A_{sub} from the image as:

$$\mathbf{A}_{sub}^{k} = \begin{bmatrix} a_{1,k} & \cdots & a_{1,n-m+k} \\ \vdots & \ddots & \vdots \\ a_{n_1,k} & \cdots & a_{n_1,n-m+k} \end{bmatrix} \tag{24}$$

Thus, two-dimensional convolution operations can be divided into m one-dimensional

convolution operations, as shown in the equation:

$$\mathbf{A} * \mathbf{W} = \sum_{k=1}^{m} A_{sub}^{k} * W^{k} \tag{25}$$

Where the W^k represents the kth column in the kernel W. Next, after being flattened, $\mathbf{A_{sub}}$ is loaded onto the light via MZMs (T.MXH1.5), and the kernel is loaded onto the frequency comb using the waveshaper. It is important to note that since the AWGR can perform parallel convolution operations, this means that more than one set of dot product computation is carried out in a single time slot. Therefore, the $\mathbf{A_{sub}}$ can be loaded onto the Arbitrary Waveform Generator (AWG) at intervals of *p-m* symbols, which can increase the computation speed by a factor of *p-m*. Here the *p* represent the channel number of the AWGR. Fig. S7 illustrates the decomposition principle when $m = 3$ and $\mathbf{W}$ is given by $\begin{bmatrix} 0 & -1 & 0 \\ -1 & 5 & -1 \\ 0 & -1 & 0 \end{bmatrix}$. It shows the results of sharpening a lighthouse photo. In this case, $\mathbf{W}$ is decomposed into $[0 \quad -1 \quad 0]^T$, $[-1 \quad 5 \quad -1]^T$,and $[0 \quad -1 \quad 0]^T$. When performing image convolution, we use max normalization to display the image, which results in insufficient contrast. Methods such as average power normalization are also employed in some other cases.

The loaded figure signal **I** and the kernels **P** can be considered that there exists the following relationship:

$$\begin{cases} \mathbf{I} = a_1 \cdot \mathbf{A} + b_1 \\ \mathbf{P} = a_2 \cdot \boldsymbol{\omega} + b_2 \end{cases} \tag{26}$$

Where the a_1 and a_2 represent the modulation depth of the signal and the b_1 and b_2 denote to the bias. The output signal, which is the convolution of *I* (the image signal) and *P* (the kernel), can be denoted as:

$$\begin{aligned} \mathbf{O} = \mathbf{I} * \mathbf{P} &= (a_1 \cdot \mathbf{A} + b_1) * (a_2 \cdot \boldsymbol{\omega} + b_2) \\ &= a_1 a_2 (\mathbf{A} * \boldsymbol{\omega}) + a_2 b_1 \boldsymbol{\omega} + b_1 b_2 + a_1 b_2 \mathbf{A} \end{aligned} \tag{27}$$

In the equation, **A** is a high-speed data, while $\boldsymbol{\omega}$ remains constant. Therefore, $a_2 b_1 \omega + b_1 b_2$ is constant, it can be directly eliminated. However, since **A** varies over time, it can distort the received signal. So, it is necessary to set b_2 to zero to eliminate this term. Additionally, since **P** is greater than zero, $\boldsymbol{\omega}$ cannot be negative values. The paper achieves the loading of negative weights by utilizing two cycles of the microring to separately load the positive and negative parts of the weight.

This strategy is detailed in Fig. S7. The final decomposition formula for this strategy is as follows:

$$\mathbf{A} * \mathbf{W} = \sum_{k=1}^{m} \mathbf{A_{sub}^{k}} * \mathbf{W^{k}} \tag{28}$$

The figure illustrates the decomposition principle when $m = 3$ and $\mathbf{W}$ is given by $\begin{bmatrix} 0 & -1 & 0 \\ -1 & 5 & -1 \\ 0 & -1 & 0 \end{bmatrix}$. It shows the results of sharpening a lighthouse photo. In this case, $\mathbf{W}$ is decomposed into $[0 \quad -1 \quad 0]^T$, $[-1 \quad 5 \quad -1]^T$,and $[0 \quad -1 \quad 0]^T$. When performing image convolution, we use max normalization to display the image, which results in insufficient contrast. Methods such as average power normalization are also employed in some other cases.

With the support of the aforementioned technologies, this approach enables parallel

task execution. We utilize a combination of pilot-based synchronization and pre-calibration to ensure signal alignment. This process may introduce a delay exceeding 10 ns, which remains acceptable in the context of image processing. We believe that the parallel approach can significantly enhance data processing speed and improve task execution efficiency.

Supplementary Note 5: Principle of Loading Both Positive and Negative Weight Based on Microring-Enhanced AWGR

The proof presented in the main text demonstrates that it is challenging to achieve negative weights using a structure solely based on AWGR. Compared to using two separate devices to achieve both positive and negative weight loading, leveraging the inherent periodic routing characteristics of the AWGR is a more straightforward method for implementing positive and negative weights. Therefore, we introduce microrings in our design to realize this feature. Microring filters are a well-established technology, known for their precise filtering of specific wavelengths and negligible insertion loss. Additionally, the filtered light can be output from another port. With these characteristics, we can easily use microrings to separate wavelengths that are adjacent by one FSR. In the AWGR, wavelengths spaced by one FSR have the same routing characteristics and can be considered as twin pairs with the same routing behaviors. Thus, they can be loaded with different weights but undergo the same computational operations.

We load the positive parts of the weights onto the wavelengths in the first cycle and the negative parts onto the wavelengths in the next cycle. In the MZM, they will experience the same modulation and therefore be loaded with the same input signal. These twin pairs can only be distinguished and separated by the microring, which directs them to the two input ports of the BPD. The signal from the first cycle's wavelength is directly converted to current output, while the signal from the second cycle's wavelength is inverted before being output and added to the signal from the first cycle's wavelength. Fig. S9 shows that the signals from the two cycles will eventually be output in the following form:

$$Output = a_1 + a_2 + a_3 - (b_1 + b_2 + b_3) \quad (29)$$

When loading weights in practice, since a single weight can only exist in either positive or negative form, if a_1 has a value, b_1 must be zero. Only one of the twin pairs can be active at any given time.

Fig. S10 illustrates the paths of different wavelengths when the structure shown in Fig. S4 is combined with microrings. Solid and dashed areas represent two different FSRs. Due to space constraints in the figure, only the transmission relationships for two sets of wavelengths are depicted. However, this is sufficient to explain how the structure supports the loading of both positive and negative weights. Unlike Fig. S4, since there are only two sets of wavelengths, the weight length is equivalent to 2. In this scenario, the output signal length is 3, which is why three output signals are highlighted in the figure.

Fig. S11 shows the filtering curve of the microring filter we designed. The reason for

using a three-ring design is to achieve a broader transmission peak. The experiments in this study utilized convolution kernels with a length of three taps, requiring the filter to cover three consecutive wavelengths. In simulations, we found that a single-ring microring filter could not achieve such a flat and sufficiently wide transmission peak. Therefore, a cascaded microring design was employed to meet this requirement.

Supplementary Note 6: OPU Convolution Accuracy Measurement

After performing the calibrations, we proceeded with testing the computational accuracy. During the accuracy tests, we transmitted a staircase signal and measured the signal-to-noise ratio (SNR) of the received signal to determine the computational accuracy. The bit-precision was derived from the standard deviation of the errors of all tested points and then converted to the expression of bits by Equation in paper[3]. The computational accuracy was calculated using Equation S22:

$$\text{bit-precision} = \log_2\left(\frac{\max(Rx(1))-\min\,(Rx(-1))}{std(Rx-Tx)}\right) \quad (30)$$

Under this scheme, previous work[1] achieved a computational accuracy of 9 bits at a working speed of 10 MHz. In this experiment, due to the precision limitations of the AWG (8 bits), a computational accuracy of 7 bits was ultimately achieved at 10 GHz.

During the precision testing process, the precision of four different computational processes is validated experimentally. Among these, addition, multiplication and subtraction involve two input signals, while multiply-accumulate (MAC) operations involve three input signals, with a total of 49152 symbols, each at 8 different power levels. In the addition experiment, the result of the addition can be detected using just one photodetector. In contrast, during the subtraction test, the two signals to be subtracted are output from the two arms of the microring. In the designed architecture, this step should be performed using a BPD. However, due to the insufficient bandwidth of our BPD, the method has been adapted to use two photodetectors to receive the signals, which are then subtracted electronically in the computer. In all the multiplication operations, one signal is loaded through an MZM, while the other is loaded via a waveshaper onto the amplitude of frequencies. These can be expressed as follows:

$$\begin{cases} Z_a = X_1 + X_2 \\ Z_s = X_1 - X_2 \\ Z_m = X_1 Y_1 \\ Z_{mac} = X_1 + X_2 Y_1 \end{cases} \quad (31)$$

Where X_i represents the signals loaded through MZMs, which are always loaded via an AWG at a sampling rate of 100-GHz. Y_i represents the signals loaded through waveshaper, typically representing constant weight signals. Za, Zs, Zm, and Zmac represent the results of addition, subtraction, multiplication, and multiply-accumulate operations, respectively.

The box plots for addition and subtraction at different baud rates are shown in Fig. S12. The area of the box plots reflects the noise distribution from the 25th to the 75th percentile. The noise introduced at higher baud rates has multiple sources. Frequency attenuation is the main factor, as the signal's high-frequency components are reduced

under large bandwidth conditions, leading to a degraded SNR for these components. Additionally, the increased signal bandwidth occupies a broader spectral range, making it more susceptible to white noise.
This design has been successfully demonstrated to integrate MZM, AWGR, MR, and PD arrays on a single silicon chip. We experimentally tested the performance of this structure after packaging, confirming its practicality. The experimental results are shown in Fig. S19, and the system's end-to-end bandwidth is expected to reach 35 GHz. In this experiment, when testing system bandwidth, both the modulator and photodiode used were on-chip components. The optical signal was fed into the AWGR directly from one modulator and subsequently received by a PD, which converted the signal back to an electrical output.

Supplementary Note 7: The Experiment of Optical Communication

The experimental setup for modulating an optical frequency comb involves a laser connected to three phase modulators and one intensity modulator (Fig. S13). A 21GHz RF signal generated by an adjustable RF source is split into four paths, three of which require phase control. After phase matching of the four signals, the RF signal is amplified by electronic amplifiers. All devices are commercial, offering the advantage of controllable frequency comb spacing compared to integrated optical combs. In the experiments, adjustments to the three phase shifters are necessary to increase the number of output comb teeth, while the bias voltage of the intensity modulator controls the flatness of the optical frequency comb.
The signal from the optical frequency comb undergoes transmission experiments through the optical path as shown in Fig. S16. The curves showing the variation of the resonant wavelength with the driving voltage of the integrated TiN heater and reversed-bias voltages are displayed in Fig. S14. The shift in the resonant wavelength is directly proportional to the power of the TiN heater and the reversed-bias voltages. Initially, the microring modulator shows an extinction ratio of 29dB at a wavelength of 1546.14nm. The TiN heater can cause significant shifts in the resonant wavelength. As the voltage increases to 3V, the resonant wavelength shifts by 1.5 nm to 1547.66 nm. Reversed-bias voltages also slightly shift the resonance peak of the microring while affecting the modulator's extinction ratio. As the voltage increases to 6V, the resonance peak moves to 1546.33 nm, but the extinction ratio decreases by 5.5-dB. The extinction ratio of the microring modulator significantly impacts the modulation depth of the signal, hence in experiments, adjusting the driving voltage of the TiN heater to match the microring's resonant wavelength with the target wavelength is primarily done.
Both reversed-bias voltages and optical bias points affect the modulation bandwidth of the microring modulator. Fig. S15 shows the transmission bandwidth curve of the entire system, where the 10 dB-bandwidth is enough for the communication system. As reversed-bias voltages increase from 0V to 5V, the system 10 dB-bandwidth increases from 40GHz to 45GHz, with the significant increase occurring from 0V to 2V. Considering the impact of reversed-bias voltages on the extinction ratio, selecting a bias of 2V is most appropriate to balance extinction ratio and bandwidth. Once the extinction ratio is set at 2V, different optical bias points can be explored to find a balance between

bandwidth and transmission performance, as shown in Fig. S15b. As the reversed-bias voltage increases, the modulation bandwidth of the microring modulator expands.

Fig. S16 presents the experimental setup of the system during a metropolitan communication trial, in which a total loss of 22 dB occurs due to device imperfections, with 12 dB attributed to the optical couplers, 6 dB to the Waveshaper, and 4 dB to the optical filter. According to the Table S5, losses can be reduced by approximately 16.7 dB under ideal conditions. To address the substantial impact of transmission distance on modulated signals, we specifically designed this experiment to assess the architecture's viability over an 80 km distance. The transmitter firstly generates an 84 GHz spaced optical frequency comb, which is then modulated using an on-chip microring modulator before being transmitted through 80 km of optical fiber. To tackle dispersion issues, the signal undergoes filtering after 80 km to retain its single sideband, which is then amplified by an optical amplifier for detection. Unlike the computation-required weights that are point frequencies and unaffected by dispersion over distance, the communication experiment uses single-sideband modulation to combat dispersion, enhancing resistance to dispersion and improving communication efficiency. This technique preserves only one sideband of the received signal, avoiding frequency chirping caused by dispersion in direct reception, thus enabling IMDD transmission to support metropolitan area network services.

Supplementary Note 8: Details and Results of Optical Cloud Computing

Fig. S17 illustrates the architecture of the optical cloud computing system. We will now discuss the details of the cloud optical computing technology and experiments. Fig. S17 a and b show the packaged and original versions of the chip, respectively. The chip comprises an 8-input, 8-output AWGR and eight microring filters, each consisting of two microrings whose resonant peak are controlled by TiN heaters. In the experiments, weights are modulated by the waveshaper, transmitted through optical fiber into the computing unit where the input is loaded onto the MZM array via an AWG. After passing through the AWGR and microring filters, the signals are amplified by an EDFA and detected by a PD.

In order to test the feasibility of the approach, the experiment utilized various discrete components, resulting in substantial losses. In the experiments, the optical link loss was as high as *18-(-10)=28 dB*, calculated from the power entering the MZM to the power output from the chip. Considering the on-chip system, this loss can be reduces to *3+3+1.1+1.45=8.55 dB*, which includes *3 dB* for the MZM insertion loss, *3 dB* for modulation bias point, *1.1 dB* for the directional coupler[4], and *1.45 dB* for the AWGR[5]. This results in a *28-8.55=19.45 dB* power budget, sufficient to support fiber transmission distances up to *19.45/0.2=97 km*, which meets metropolitan area network requirements. Subsequent results confirmed that the same link loss does not affect the accuracy of image computations, supporting the robustness of our optical computing approach.

In optical computing based on AWGR, several challenges were encountered: channel-to-channel loss variability in the AWGR, alignment issues between the AWGR's transmission peaks and the optical comb, and both linear and non-linear losses in the

system. Fig. S17 d demonstrates how calibration is performed.
To verify the impact of transmission distance on computational performance, we conducted experiments based on the setup in Fig. S14 to study the influence of total loss on computational efficiency, using Peak Signal-to-Noise Ratio (PSNR) to evaluate output performance. The length of the fiber changes from 0km to 80 km. As shown in Fig. S18a, as the loss increases, the PSNR initially remains stable but then rapidly decreases. This stability at attenuations below 8 dB is due to the sufficient optical power. As attenuation continues to increase, performance deteriorates more rapidly due to the influence of receiver noise as optical power decreases. Fig. S18b confirms that our optical computing approach is unaffected by the fiber transmission distance. In our experiments, the total link loss is fixed at 16 dB and the PSNR of the computational output is recorded as the fiber length varied. The PSNR only drops from 32.59 to 32.53 dB as the transmission distance increases from 0 km to 80 km.
Fig. S19 illustrates the system performance after packaging the MZM, AWGR, microring, and PD array on the same chip. We evaluated the signal eye diagrams and bit error rates at various baud rates for end-to-end processing.
MNIST images are processed through a convolutional layer with a kernel size of 3x3, padding of 0, stride of 1, and 6 output channels, as shown in Fig. S21. This is followed by ReLU activation, a 2x2 max-pooling layer, and then the data is flattened into a one-dimensional vector. Finally, a fully connected layer outputs the probabilities that the image belongs to one of the 10 classes (digits 0 to 9). Gaussian white noise with 5-bit quantization is added after each layer to simulate the computational noise of the optical chip.
The testing process is divided into three steps. First, the model is trained offline with noise added. Second, 120 images are randomly selected from the test set, and the convolutional layer computations are deployed onto the optical chip, while the remaining computations are carried out offline. Third, the model is fine-tuned using 1/6 of the data (20 out of 120 images), and the classification accuracy on the remaining 100 images is taken as the final experimental result.
The overall network architecture diagrams of pix2pix[6] and CycleGAN[7] are shown in Fig. S20. The pix2pix network primarily utilizes a conditional generative adversarial network architecture. The generator receives an input image and maps it to a generated image, while the discriminator takes both the generated and real images, concatenates each with the input image, and then predicts whether they are real or fake. The generator tries to produce images that closely resemble the real ones, while the discriminator works to distinguish between the generated and real images. This adversarial process continues until the generator achieves high-quality output. The pix2pix structure is mainly used for training and testing on paired datasets.
Considering the challenges of obtaining paired data and the practical utility of generative models, CycleGAN extends pix2pix structure by employing two GAN structures that train collaboratively. Generator G maps images from domain X to domain Y, while generator F maps images from domain Y back to domain X. In addition to the original GAN loss, CycleGAN introduces a cycle consistency loss and identity loss, ensuring that the two generators can perform inverse mappings between the two

domains, enabling them to achieve mutually reversible transformations. The network architecture used in the experiments is detailed in Table S3, which provides comprehensive information on the layer composition, parameter numbers, and computational costs for each module. Additionally, the source code is available in the supplementary materials for further reference and reproducibility.

Fig. S22 and Fig. S23 display the output results of 100 handwritten digits after convolution with kernels 1 and 3, all processed at a computing speed of 50GHz. When displaying the images, the data were first quantized and normalized to a range of 0-255. The figures reveal some transmission errors in the computational results. Fig. S24 and Fig. S25 show the experimental results of the image convolution processing, including the output waveforms and the recovered images. Some other image generation results for different tasks are also shown in Fig. S26, such as object generation, mapping aerial photos, semantic segmentation and image depth detection.

In the aforementioned task, all convolution layers for handwritten digit recognition are implemented optically in the experiment. In contrast, for generative AI, only the first convolutional layer is processed in the optical domain, which is owing to the significantly larger model scale compared to the digit recognition task. To obtain enough results in our experiment, we began by numerically modeling our proposed architecture and employed this model to replace selected portions of the neural network during evaluation. This method integrates seamlessly with our proposed system. This is because our cloud optical computing architecture fundamentally aims to decompose a complex neural network computation into a sequence of individual operations. Within this framework, our device is employed to carry out discrete convolutional tasks. As a result, whether the computation originates from a single-layer or multi-layer network becomes essentially irrelevant in terms of how the operations are executed.

To verify whether the performance remains consistent after multi-layer optical domain computation, more analysis is conducted as illustrated in Fig. S27. The output results of the generative AI model were evaluated under settings with multi-layer optical-domain computations, in addition to different quantization noise levels in the electrical domain. A comparison of the results demonstrates a high degree of consistency in the outputs produced by optical-domain computations and the electrical domain with 7-bits. The results obtained from executing the first and second layers of the model in the optical domain demonstrate a performance level comparable to that of the electronic counterpart with 7-bit quantization. In the main manuscript, we examined the precision of fundamental operations and found that each maintained a computational accuracy of approximately 7 bits. This aligns closely with the results observed in Fig. S27. When only the first layer is computed in the optical domain, the model achieves the FID and LPIPS of 11.64 and 0.0201, separately. These metrics remain highly consistent after extending the optical computation to multi-layers. Specifically, the FID slightly degrades to 13.09, and the LPIPS increases marginally to 0.0210—both changes being minimal. Moreover, the simulated results obtained from optical-domain computation closely match those of the 7-bit quantized all-electrical baseline shown in the table. This consistency holds for both the single-layer and multi-layer optical computing scenarios, indicating that the outputs from optical computation are comparable to those achieved

under 7-bit quantization in electrical domain. These findings support two key conclusions: first, the inherent computational precision of optical-domain operations is approximately 7 bits; second, the performance of a network with a single optical layer can be extrapolated to networks with multiple optical layers.

To further explore the potential of optical computing for advanced and complex models, additional research has been conducted to determine whether the precision of optical computing can adequately support modern deep neural networks. Direct application of the post-training quantization method struggles to meet the requirements of large models under relatively low-precision operations. In response, many advanced algorithms[8–11] proposed to achieve low-loss quantization of large models. These quantization algorithms have typically been validated at 4, 6, and 8 bits, with the lowest quantization precision reaching 1-2 bits. Therefore, considering advanced quantization algorithms can significantly enhance the efficiency and performance of large model quantization computations. We employed the QNCD quantization method[12,13] to test the DDIM model. The results, as shown in Fig. S28 indicate that with the aid of QNCD, even at lower bit widths (such as 8 bits), the generative performance of the model is largely preserved, which is similar to the output performance supported by our device. This efficient computation at low bit widths provides a feasible implementation path, further validating the feasibility of optical computing chip systems in handling large-scale, complex generative AI models. Thus, through the synergistic optimization of hardware and quantization algorithms, it is expected that this system can efficiently operate larger scale, more complex modern deep learning models on edge devices. These advancements significantly bolster our confidence in optical cloud computing, leading us to believe that it will integrate more closely with people's lives in the future and provide a foundation for the widespread application of generative models.

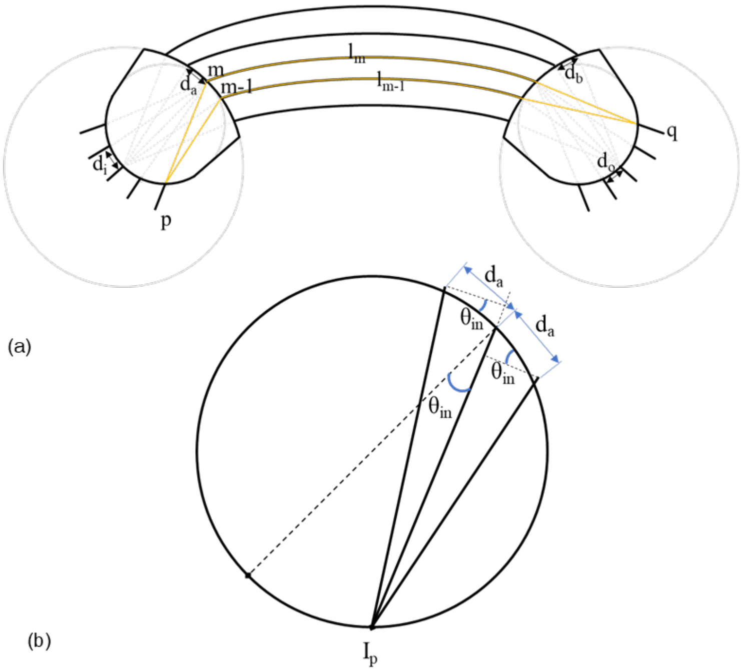

Fig. S1 AWGR Design Principles. a, Structure of the AWGR, consisting of two star couplers and a waveguide array. **b,** Diagram of light transmission paths in the star couplers. It represent the smaller circle in the star coupler.

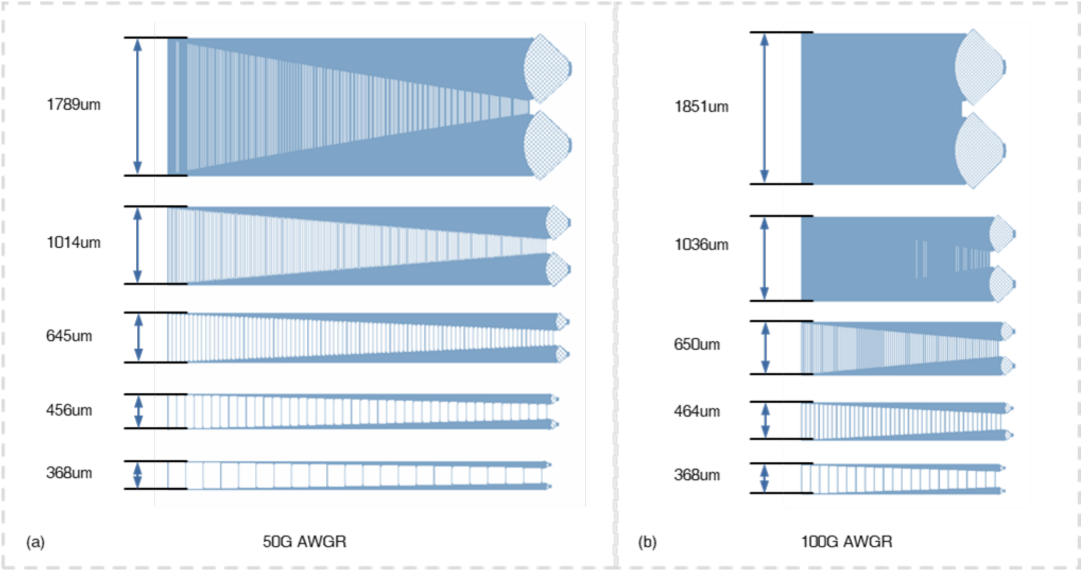

Fig. S2 Design size of AWGR for different wavelength spacings and channel numbers. From bottom to top: 4, 8, 16, 32, and 64 channels.

Table S1 Device size and the peak computational speed of the AWGR versus channel numbers with the channel spacing of 100GHz

Ch. Number	Ch. Spacing	Max Input	Max Kernel	Device Width	Computations / Clock Cycle
4	100 GHz	4	4	368um	64
8	100 GHz	8	8	464 um	256
16	100 GHz	16	16	650 um	1024
32	100 GHz	32	32	1036 um	4096
64	100 GHz	64	64	1851 um	16384

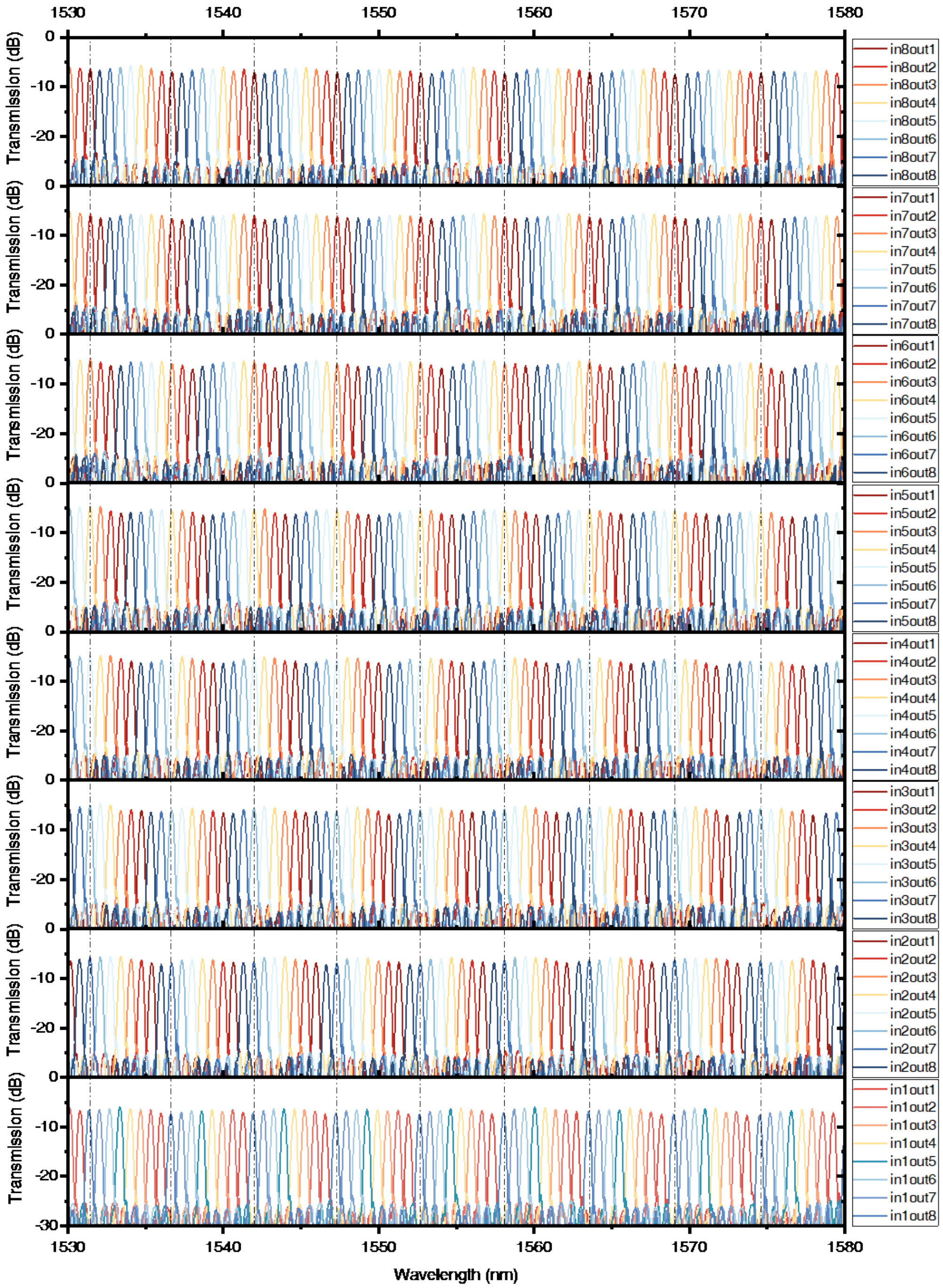

Fig. S3 Transmission spectra between the input and output ports of an 8x8 AWGR.

Table S2 Output optical power varies with changes in input and output ports.

		In1	In2	In3	In4	In5	In6	In7	In8
Output Power (dBm)	Out1	-30.5	-30.1	-29.9	-29.7	-29.7	-29.9	-30	-30.5
	Out2	-30.1	-29.8	-29.5	-29.2	-29.2	-29.4	-29.6	-30.1
	Out3	-29.9	-29.5	-29.1	-28.8	-28.9	-29.1	-29.4	-29.8
	Out4	-29.7	-29.2	-28.8	-28.7	-28.7	-28.8	-29.1	-29.5
	Out5	-29.7	-29.2	-28.8	*-28.7*	-28.7	-28.8	-29.1	-29.6
	Out6	-29.9	-29.5	-29.1	-28.9	-28.9	-29	-29.1	-29.8
	Out7	-30.1	-29.8	-29.4	-29.3	-29.2	-29.4	-29.6	-30
	Out8	-30.6	-30.2	-29.9	-29.8	-29.7	-29.8	-30	-30.5

	1	2	3	4	5	6	7	8
8	λ_1	λ_2	λ_3	λ_4	λ_5	λ_6	λ_7	λ_8
7	λ_8	λ_1	λ_2	λ_3	λ_4	λ_5	λ_6	λ_7
6	λ_7	λ_8	λ_1	λ_2	λ_3	λ_4	λ_5	λ_6
5	λ_6	λ_7	λ_8	λ_1	λ_2	λ_3	λ_4	λ_5
4	λ_5	λ_6	λ_7	λ_8	λ_1	λ_2	λ_3	λ_4
3	λ_4	λ_5	λ_6	λ_7	λ_8	λ_1	λ_2	λ_3
2	λ_3	λ_4	λ_5	λ_6	λ_7	λ_8	λ_1	λ_2
1	λ_2	λ_3	λ_4	λ_5	λ_6	λ_7	λ_8	λ_1

Fig. S4 Routing rules between different ports. Wavelengths 1-8 represent wavelengths arranged consecutively in a single FSR range. The figure illustrates the grouping mode when the wavelength entering from port 1 and exiting from port 8 is named as Wavelength 1. Under this configuration, Wavelengths 1-3 can be used for loading weights.

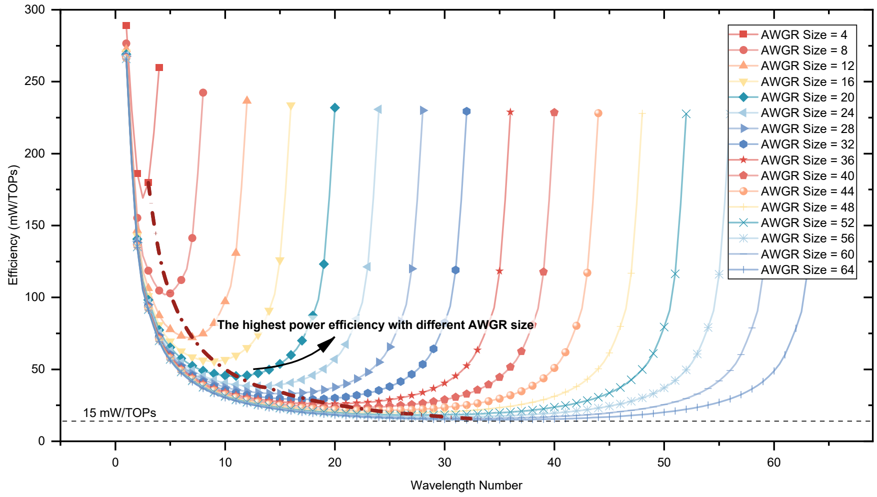

Fig. S5 The computational efficiency varies with changes in the AWGR size and the number of wavelengths employed. Generally, a larger AWGR scale can achieve higher computational efficiency, provided the number of wavelengths utilized remains within an optimal range. Beyond this range, the efficiency gains diminish. Additionally, the incremental improvement in efficiency resulting from continuously increasing the AWGR scale exhibits diminishing returns due to marginal effects

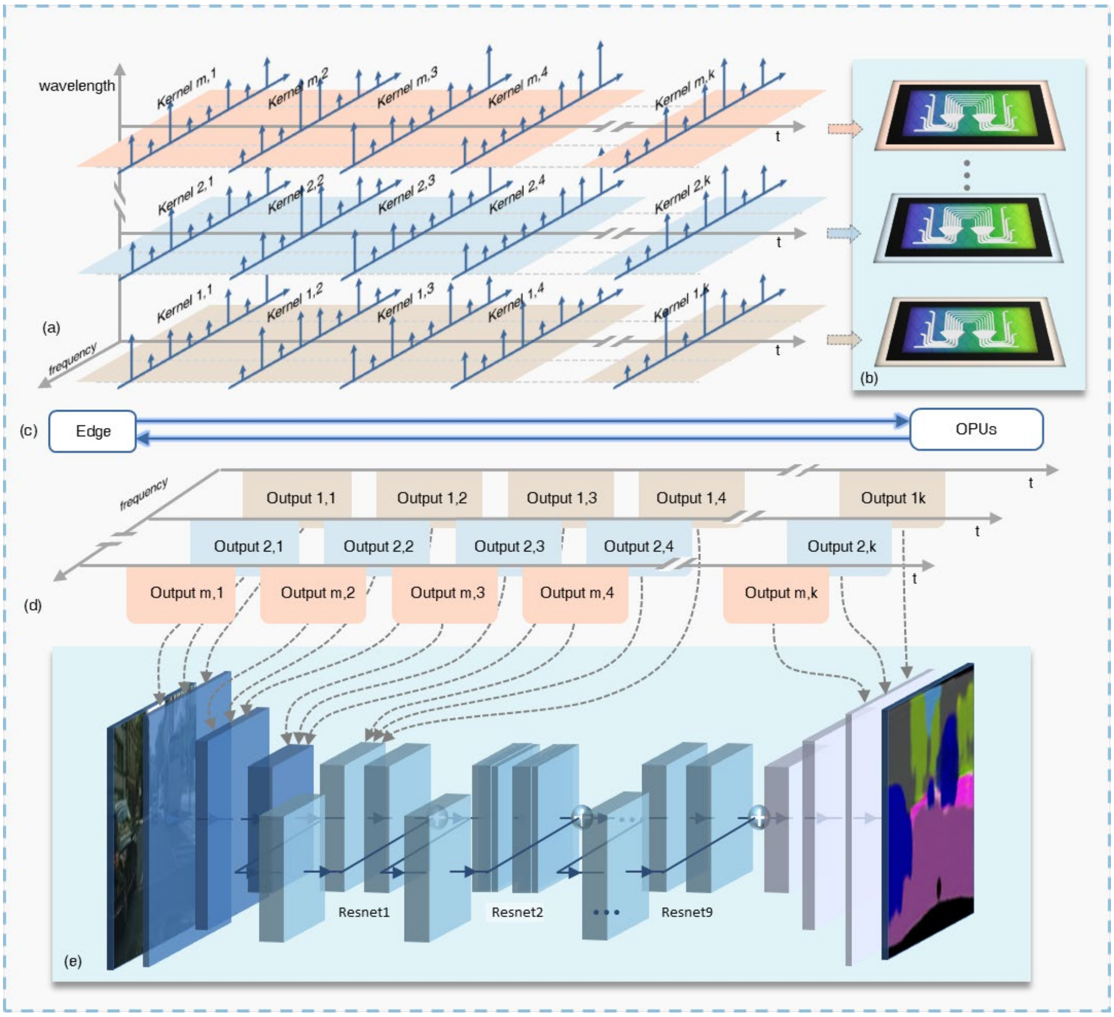

Fig. S6 Parallelization strategy in cloud optical computing. a, Weights are loaded across three dimensions (wavelength, frequency, and time), enabling multi-layer neural network computations and parallel processing. **b,** Multiple OPUs in the computing center execute distinct computational tasks. **c,** Weights and input data are transmitted from edge nodes to the computing center, while computational outputs are continuously aggregated and returned to end-users. **d,** Outputs of parallel computations are sequentially delivered to edge nodes via frequency-division multiplexing, with different time slots corresponding to neural network outputs at distinct layers in **e.**

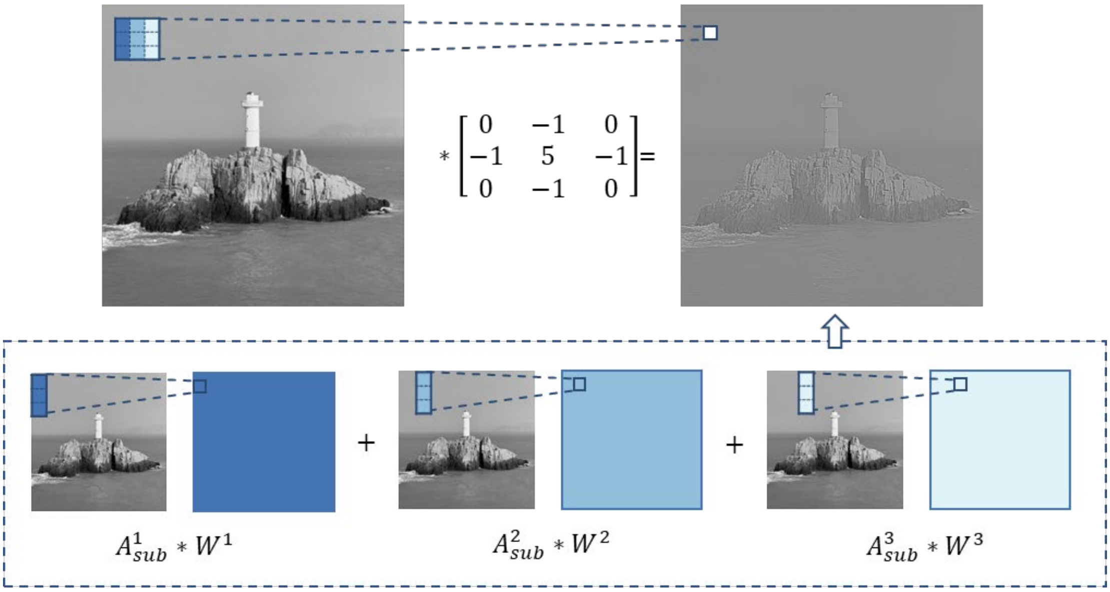

Fig. S7 Schematic diagram illustrating the principle of mapping a two-dimensional matrix convolution to several one-dimensional convolutions.

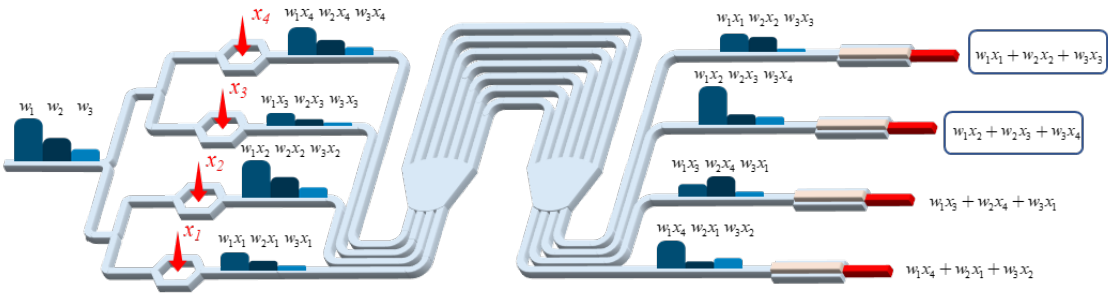

Fig. S8 Schematic of optical convolution computation within a single FSR range of the OPU. The diagram demonstrates the convolution of a signal of length 4 with a kernel of length 3. Utilizing a 4x4 AWGR as the core, the OPU performs the convolution using three wavelengths. The signal is loaded through the MZM array on the left and output through the PD array on the right. The length of the output signal is calculated as 4-3+1=2, sequentially exiting from output ports 1 and 2.

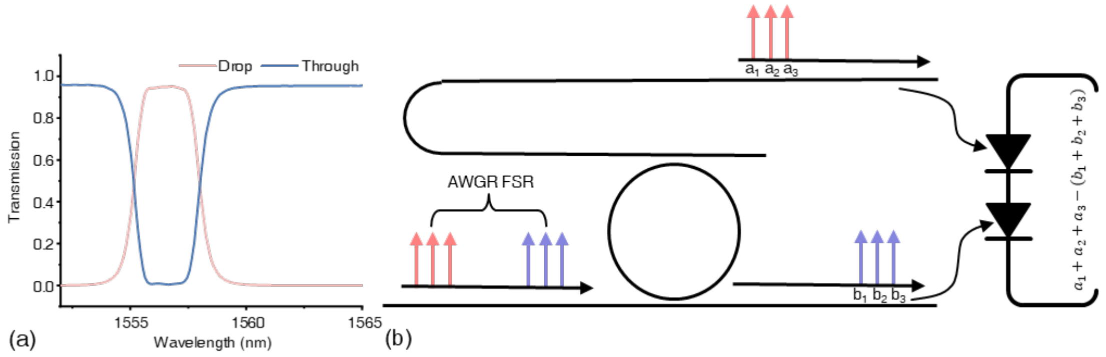

Fig. S9 Microring filter module capable of both positive and negative convolution. a, Simulated transmission curve of the microring filter's drop and through ports. **b,** Integration of the microring with the Balanced Photodetector. This setup allows signals from two FSR ranges to serve as the positive and negative values of the output signal.

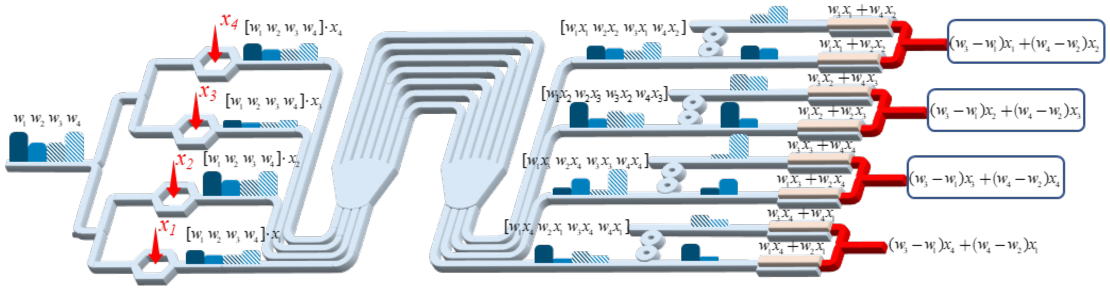

Fig. S10 Schematic of optical convolution computation within two FSR ranges of the OPU. The diagram demonstrates the convolution of a signal of length 4 with a kernel of length 2. Utilizing a 4x4 AWGR as the core, the OPU performs the convolution using four wavelengths. The signal is loaded through the MZM array on the left and output through the PD array on the right. The length of the output signal is calculated as 4-2+1=3, sequentially exiting from output ports 1, 2 and 3.

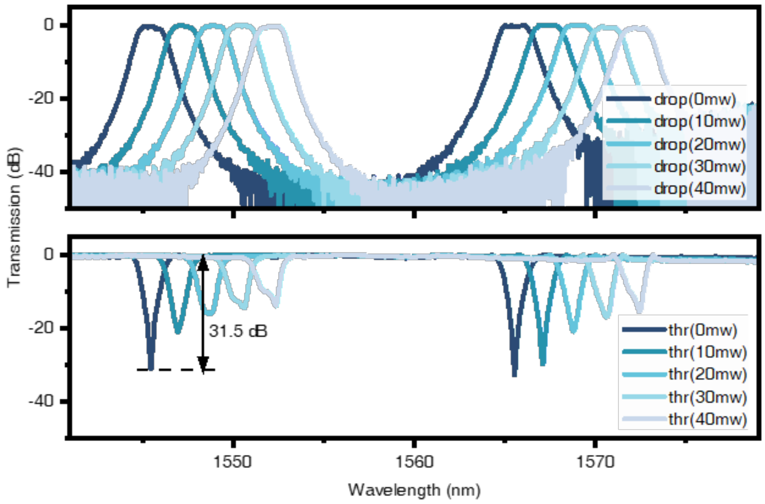

Fig. S11 Experimental results showing the variation in the transmission spectrum of the microring filter with changes in heater power.

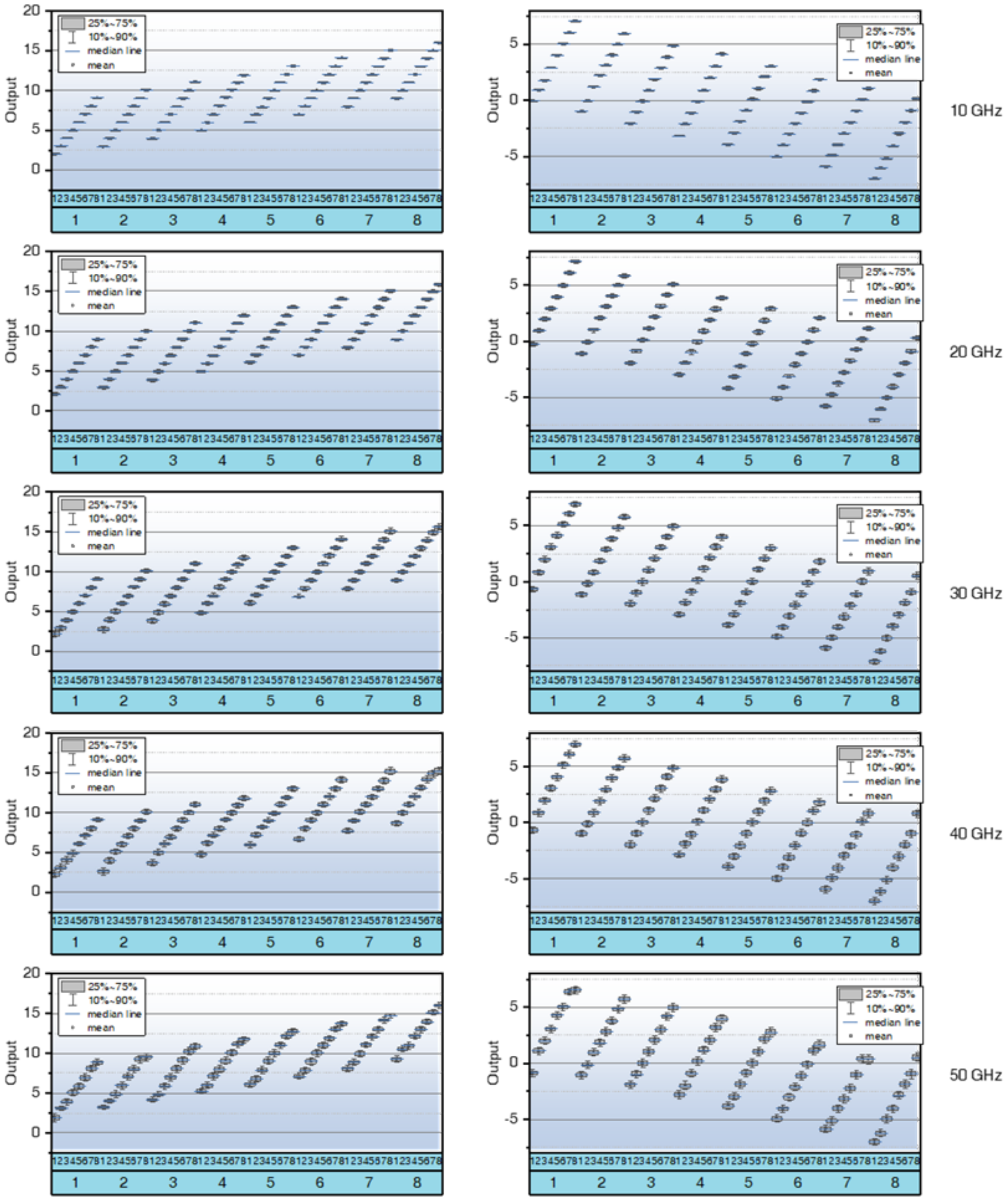

Fig. S12 Graph of computational accuracy for addition and subtraction operations at different computation speed. The highest computational accuracy occurs at a computing speed of 10GHz. It is observed that at a computing speed of 10GHz, signals within the 25%-75% range are concentrated near the median and mean lines. However, at a computing speed of 50GHz, the noise in the signals significantly increases.

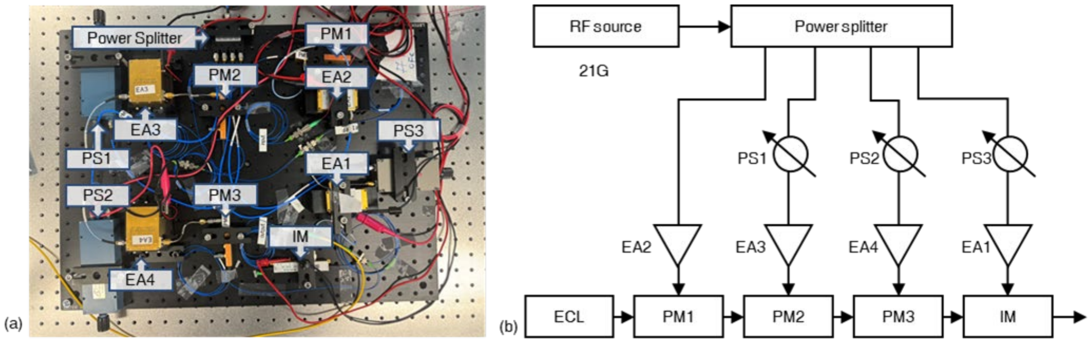

Fig. S13 Electro-optic frequency comb setup. a, Photograph of the optical frequency comb generator. PS: Phase shifter; EA: electrical amplifier; PM: phase shifter; IM: intensity modulator b, Schematic of the optical frequency comb generator.

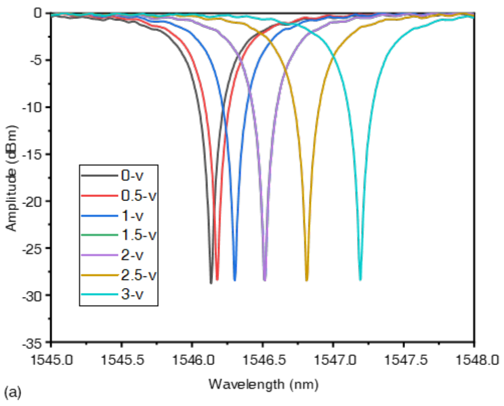

Fig. S14 a, variation of the resonant wavelength with the driving voltage of the integrated TiN heater. **b,** variation of the resonant wavelength with the reversed-bias voltages.

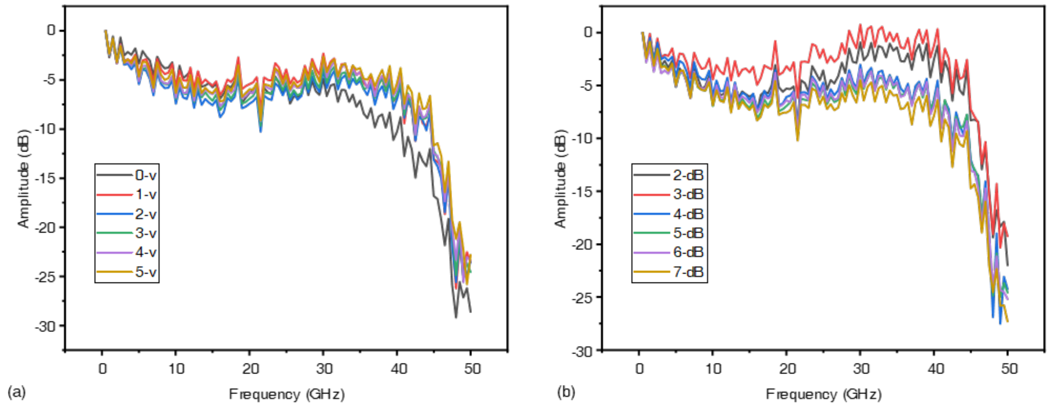

Fig. S15 a, variation of the the transmission bandwidth curve of the entire system with the reversed-bias voltages. **b,** variation of the the transmission bandwidth curve of the entire system with the optical bias points

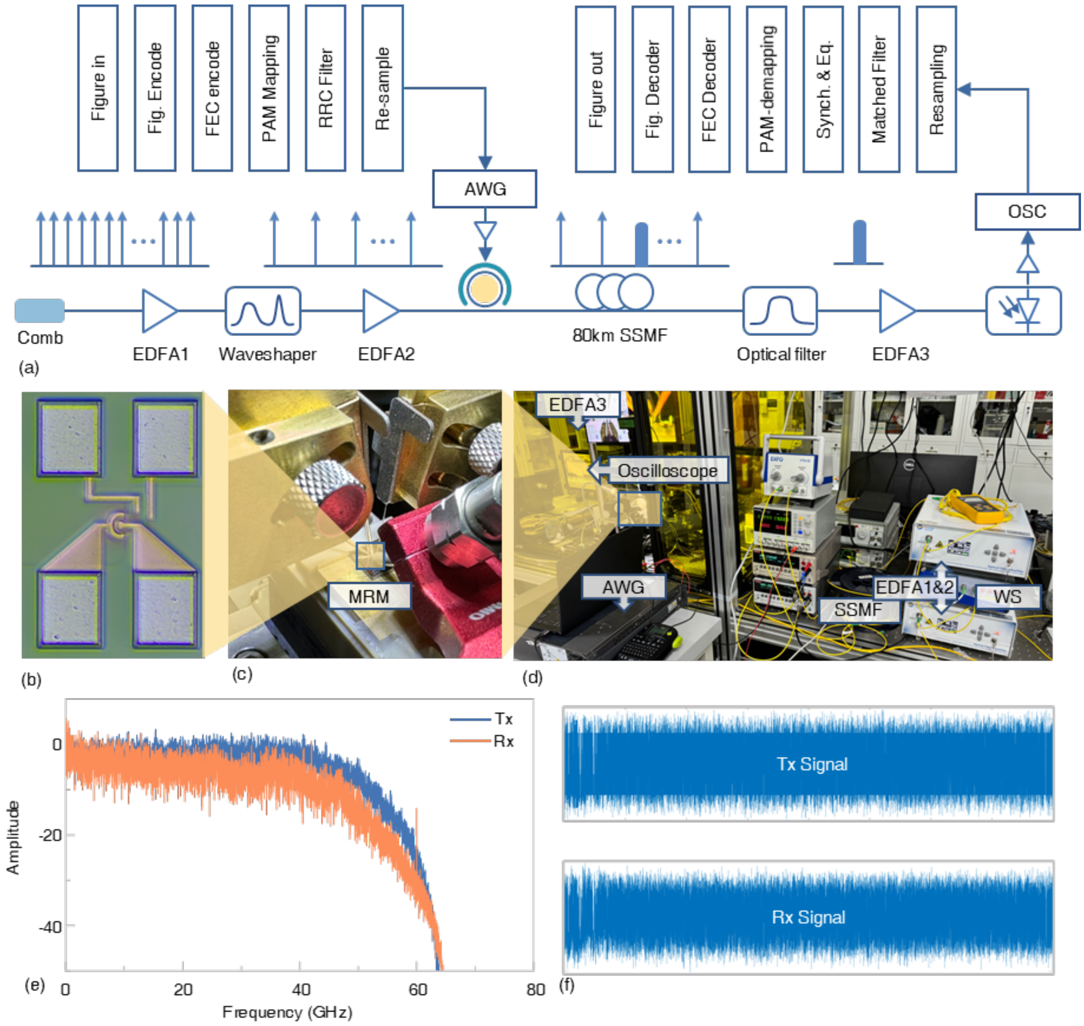

Fig. S16 Experimental demonstration of the downstream mode in optical interconnects. a, Block diagram of the experimental architecture in downstream mode. EDFA1 and EDFA2 are gain flattened amplifiers that can amplify multiple wavelengths within the C-band simultaneously while maintaining consistent gain across wavelengths, with a gain flatness of ≤1.5dB. This enables the system to operate across the entire C-band spectrum. The waveshaper supports 1GHz Grid spectral control precision, ensuring that all frequencies maintain the same power level. After passing through the fiber, the signal is received by the OPU, where an optical filter is used to filter out the signal frequency and amplify it to 5dBm. b-d, The photos of the experimental setup: the signal is loaded onto the on-chip microring modulator in (b) via the RF probe shown in (c). e-f, The changes in the transmitted and received signals in both the frequency and time domains are displayed, respectively.

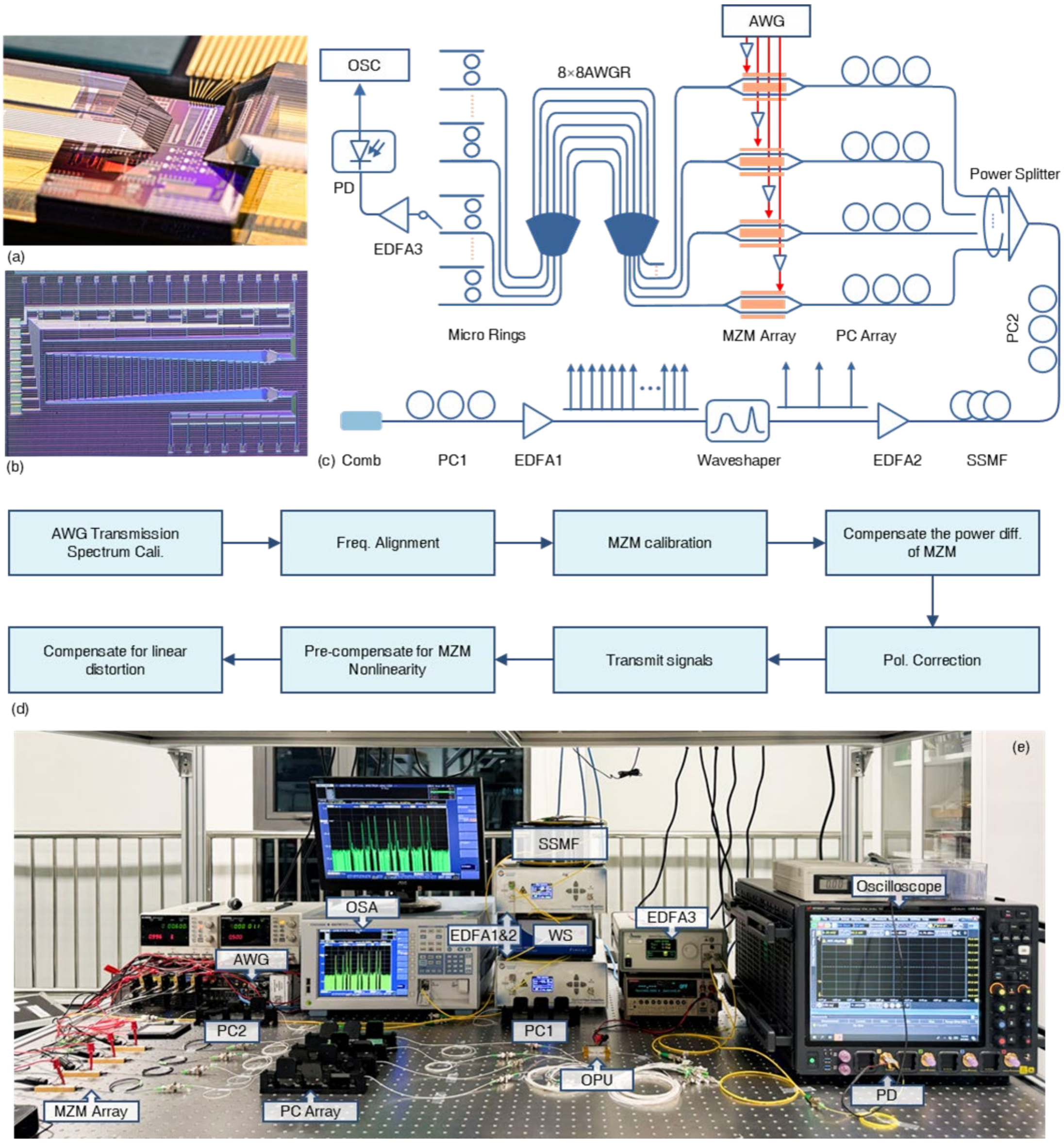

Fig. S17 Testing the computational performance of the OPU. a, Photograph of the device after packaging. **b,** Microscopic structure of the packaged chip, including the AWGR and microring array. **c,** Experimental setup where EDFA1 and EDFA2 are gain flattened EDFAs. **d,** Experimental calibration processes. **e,** Photograph of the experimental setup, where all components shown in c are mapped in e. The OPU includes an 8x8 AWGR and an array of microrings.

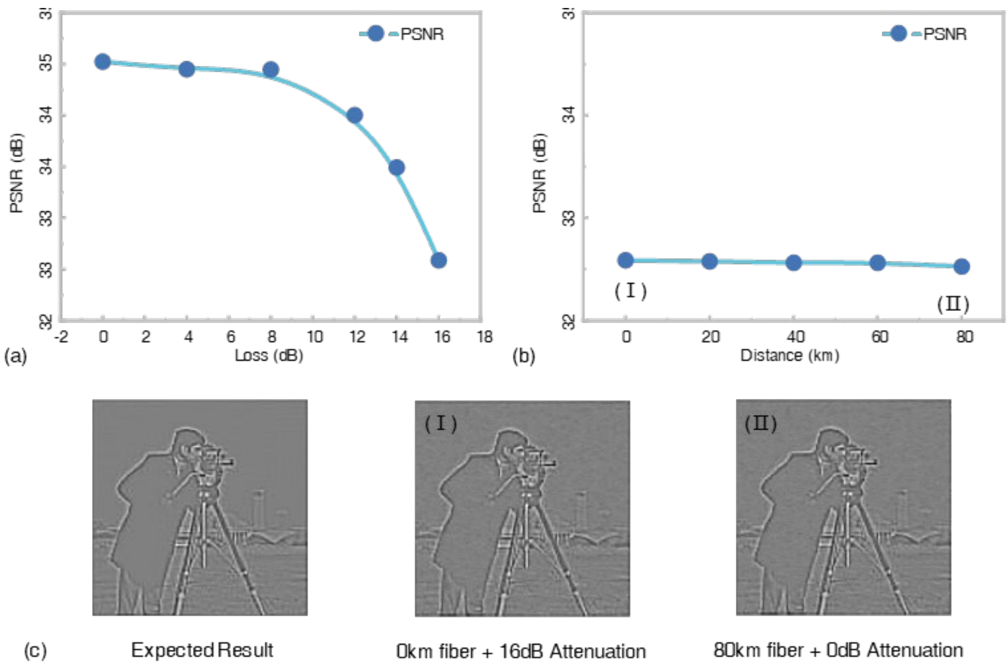

Fig. S18 Relationship between long-distance optical computing performance, loss, and transmission distance. a, The curve of optical computing performance as a function of transmission loss. Greater loss results in lower PSNR of the processed images. **b,** Variation of optical computing performance with fiber length under the same total loss. **c,** The output figures under different conditions.

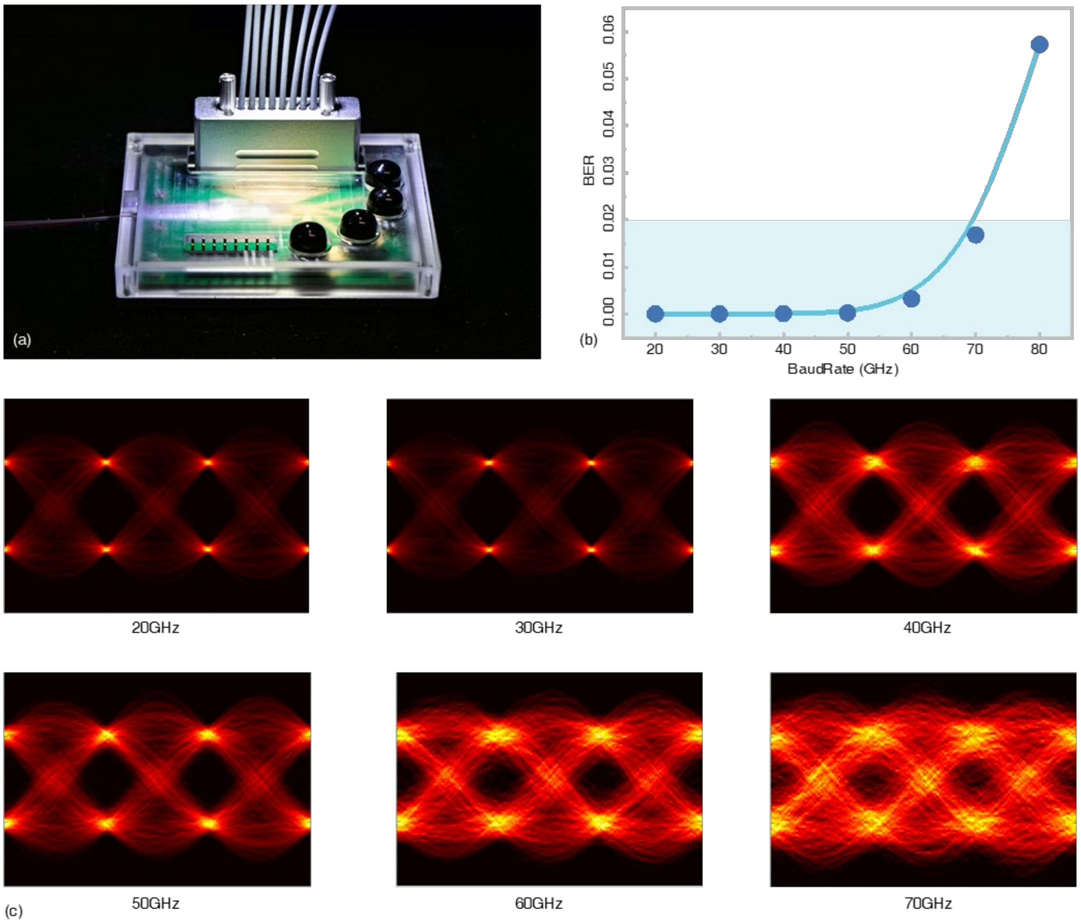

Fig. S19 Test results of the packaged OPU a, Photo of the packaged OPU, including MZM, AWGR, MR, and PD. **b,** The BER as a function of system bandwidth. **c,** Eye diagrams at different bandwidths.

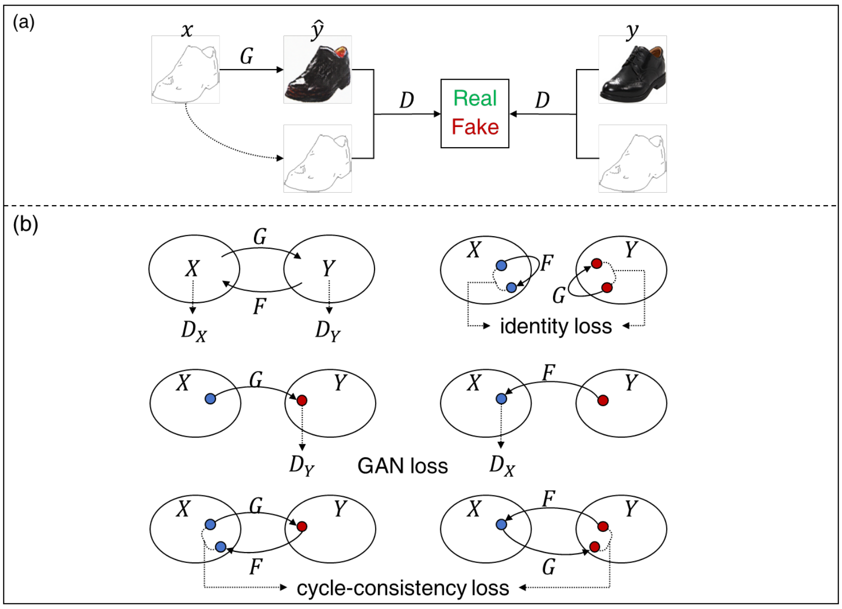

Fig. S20 The overall network architecture diagrams. a, pix2pix, **b,** CycleGAN architecture and loss function schematic.

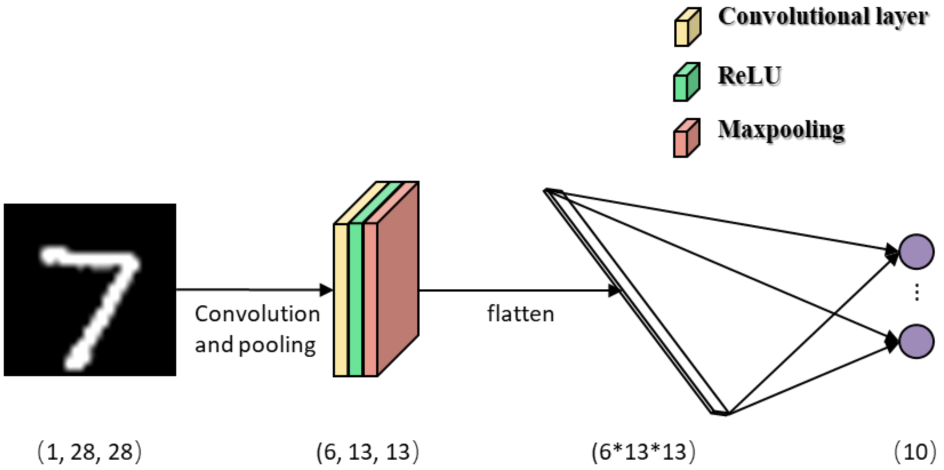

Fig. S21 Network Architecture of the MNIST images classification

Table S3 Network Architecture and Detailed Parameters. This network consists of 1 input/output module, 2 downsampling/upsampling modules, and 8 residual modules.

Module	Network Layers	Parameters	Computational cost(FLOPs)
**Input**	ReflectionPad2d(1)	/	/
	Conv2d(3,64,3,1,0)	1,792	226.49M
	InstanceNorm2d(64)	/	25.17M
	ReLU()	/	/
**DownSampling No. 1**	Conv2d(64,128,3,2,1)	73,856	2,415.92M
	InstanceNorm2d(128)	/	12.58M
	ReLU()	/	/
**DownSampling No. 2**	Conv2d(128,256,3,2,1)	295,168	2,415.92M
	InstanceNorm2d(256)	/	6.29M
	ReLU()	/	/
**Resnet Block ×8**	ReflectionPad2d(1)	/	/
	Conv2d(256,256,3,1,0)	590,080*8	4,831.84M*8
	InstanceNorm2d(256)	/	6.29M*8
	ReLU()	/	/
	ReflectionPad2d(1)	/	/
	Conv2d(256,256,3,1,0)	590,080*8	4,831.84M*8
	InstanceNorm2d(256)	/	6.29M*8
**UpSampling No. 1**	ConvTranspose2d(256,128,3,2,1,1)	295,040	9,663,68M
	InstanceNorm2d(128)	/	12.58M
	ReLU()	/	/
**UpSampling No. 2**	ConvTranspose2d(128,64,3,2,1,1)	73,792	9,663,68M
	InstanceNorm2d(64)	/	25.17M
	ReLU()	/	/
**Output**	ReflectionPad2d(1)	/	/
	Conv2d(64,3,3,1,0)	1,731	226.49M
	Sigmoid()	/	/
**Total**	/	10,182,659	102.10G

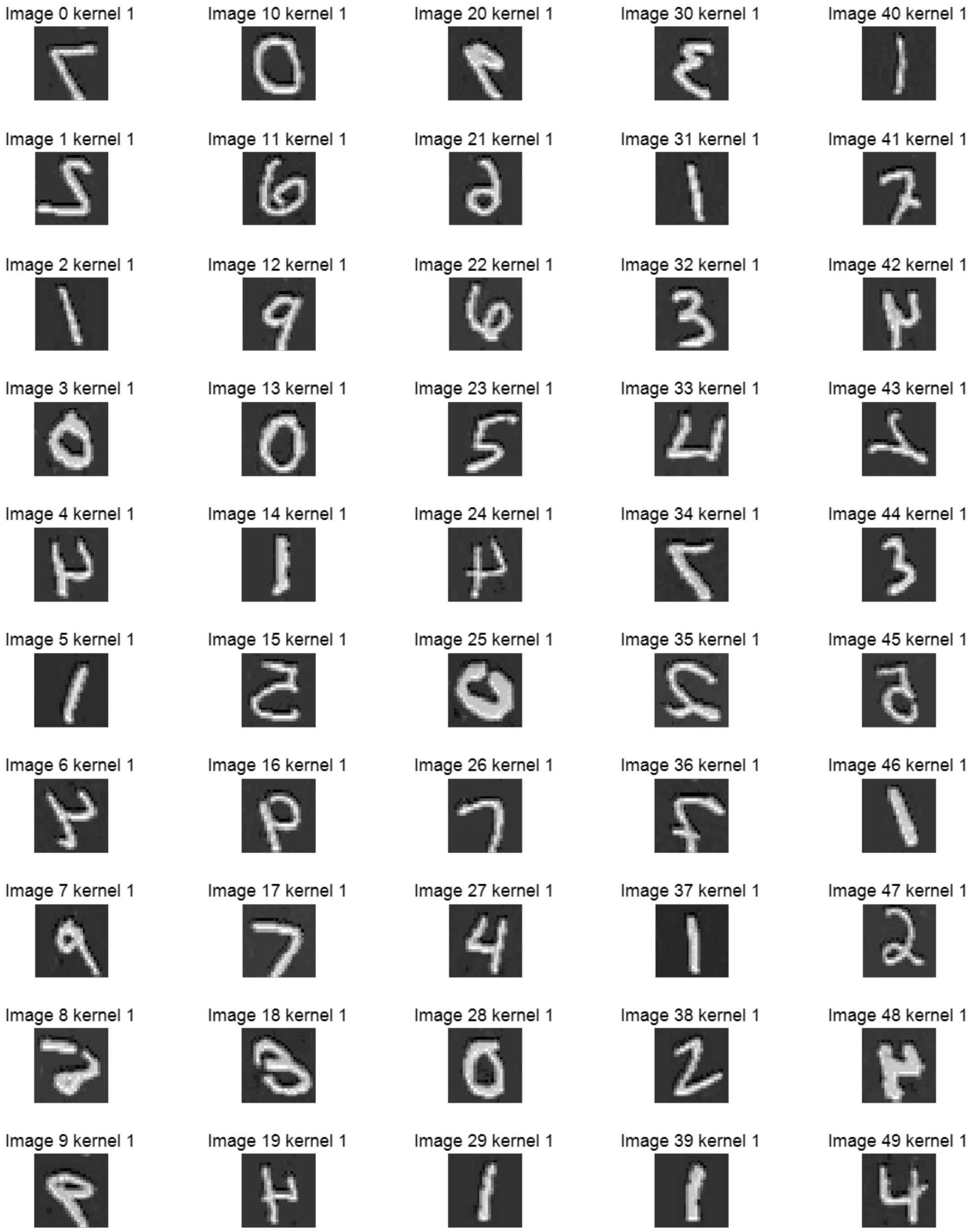

Fig. S22 Output images of MNIST handwritten digital image 0-49 after convolution with kernel 1.

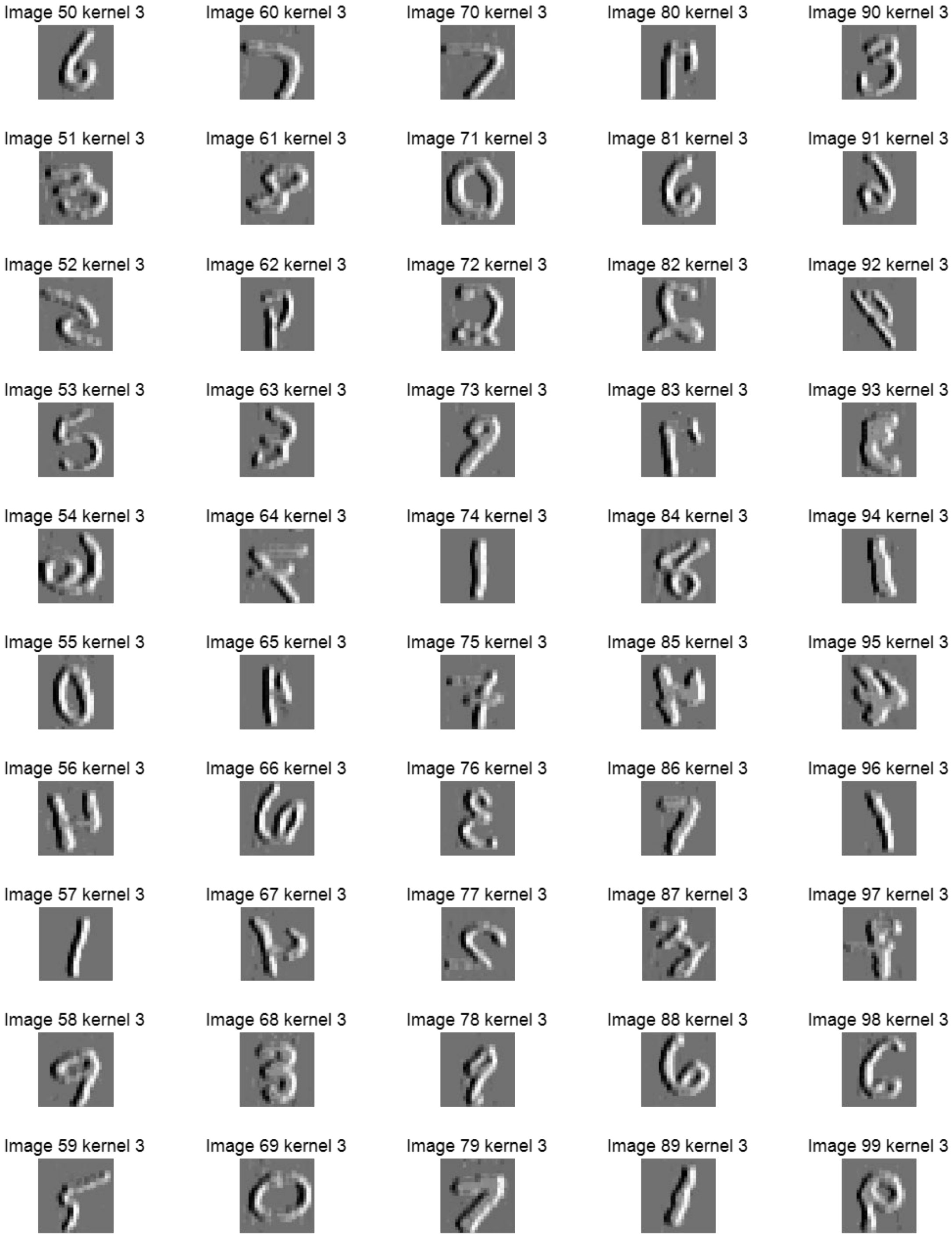

Fig. S23 Output images of MNIST handwritten digital image 50-99 after convolution with kernel 3.

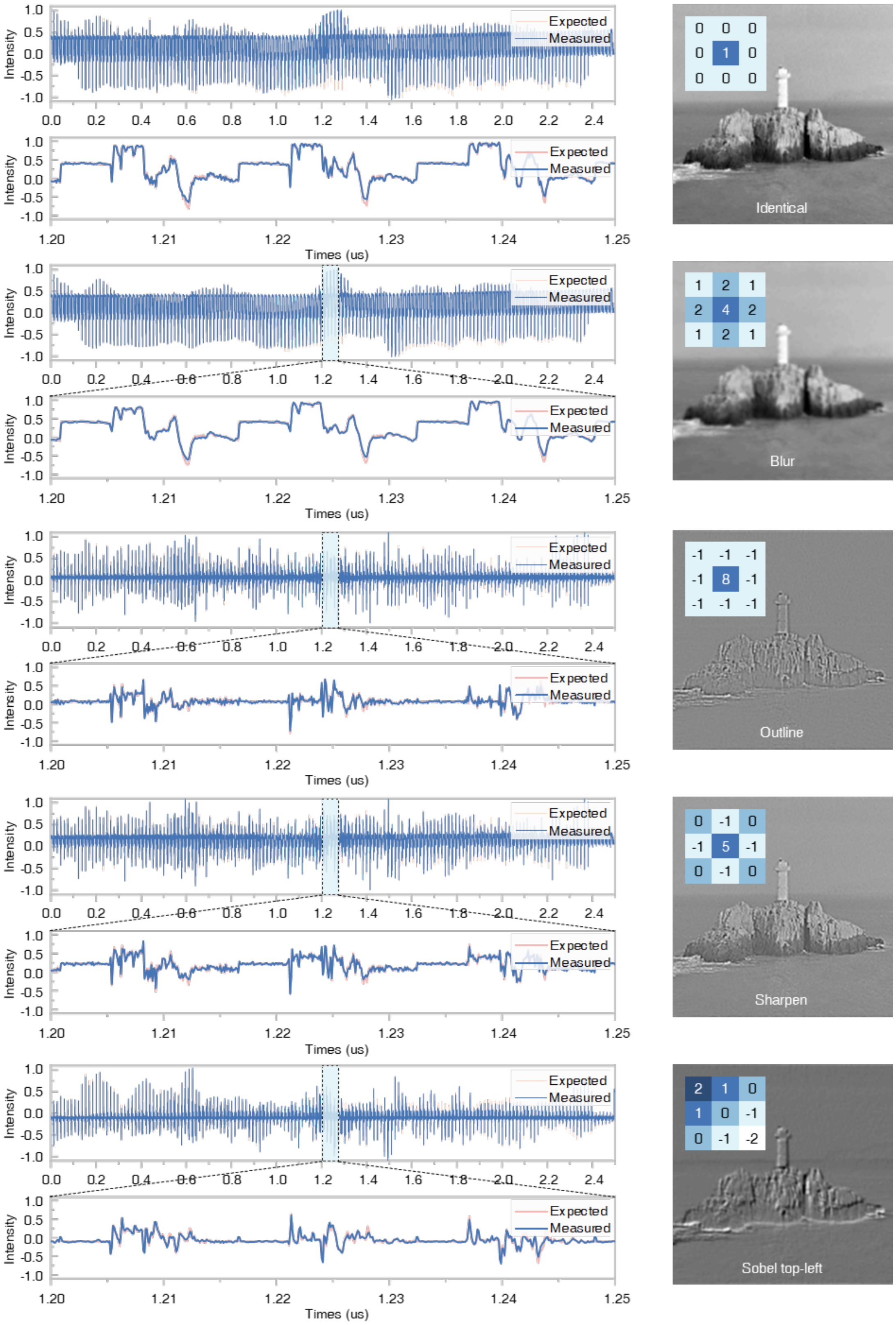

Fig. S24 The experimental and expected theoretical results from the image convolution processed by the OPU using the first 5 kernels.

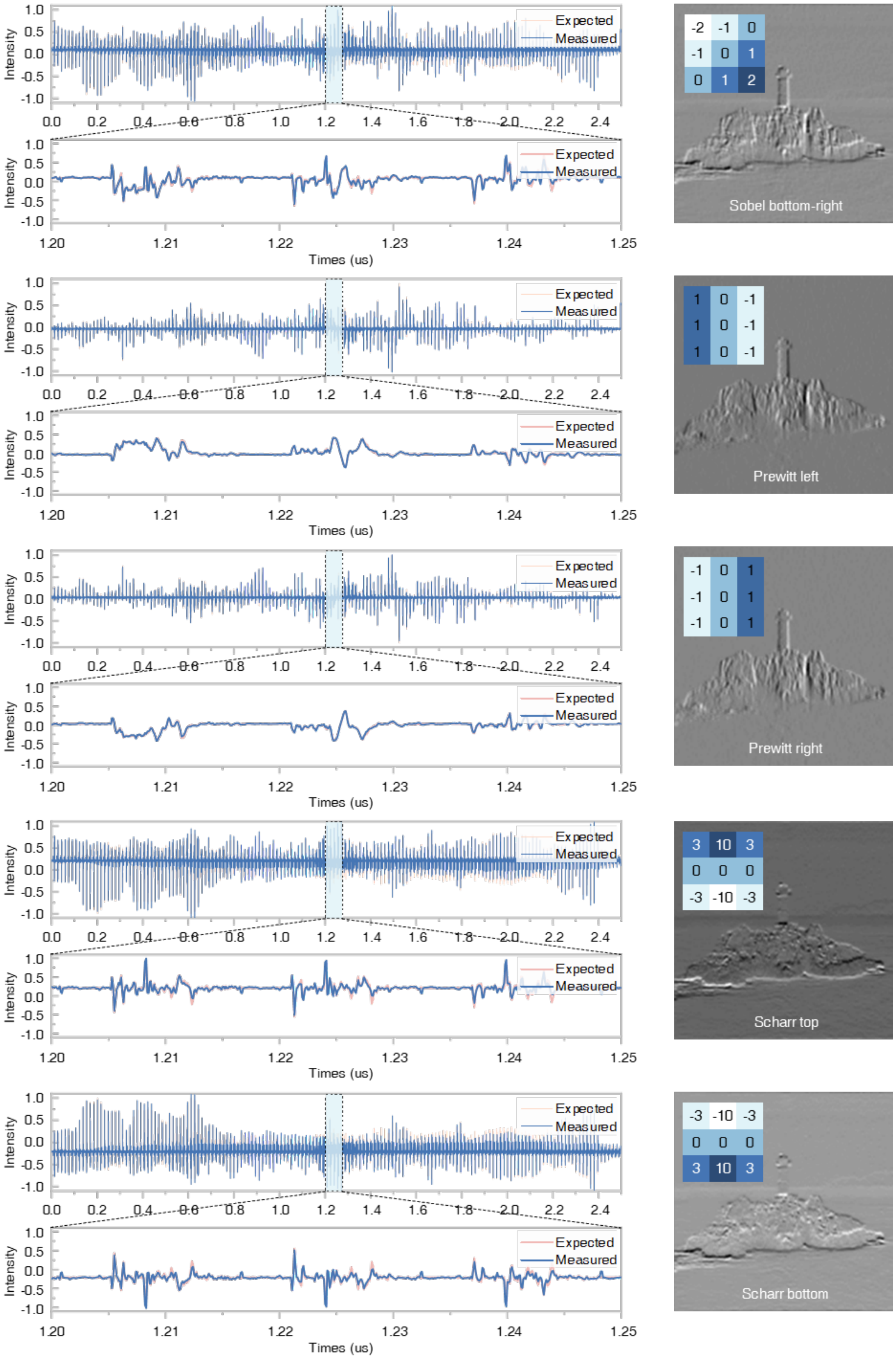

Fig. S25 The experimental and expected theoretical results from the image convolution processed by the OPU using the second 5 kernels.

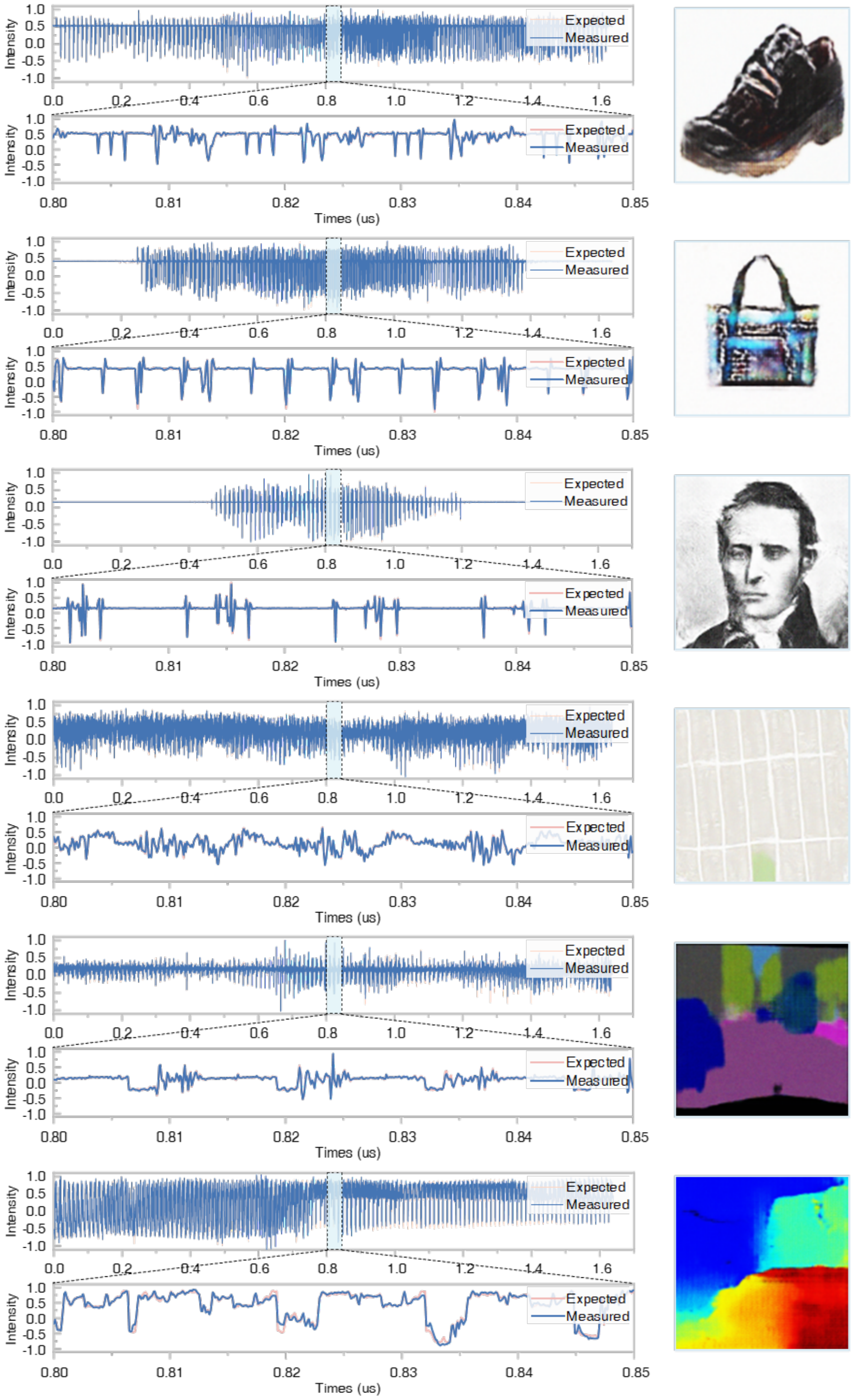

Fig. S26 Image generation results for different tasks, object generation, mapping aerial photos, semantic segmentation and image depth detection

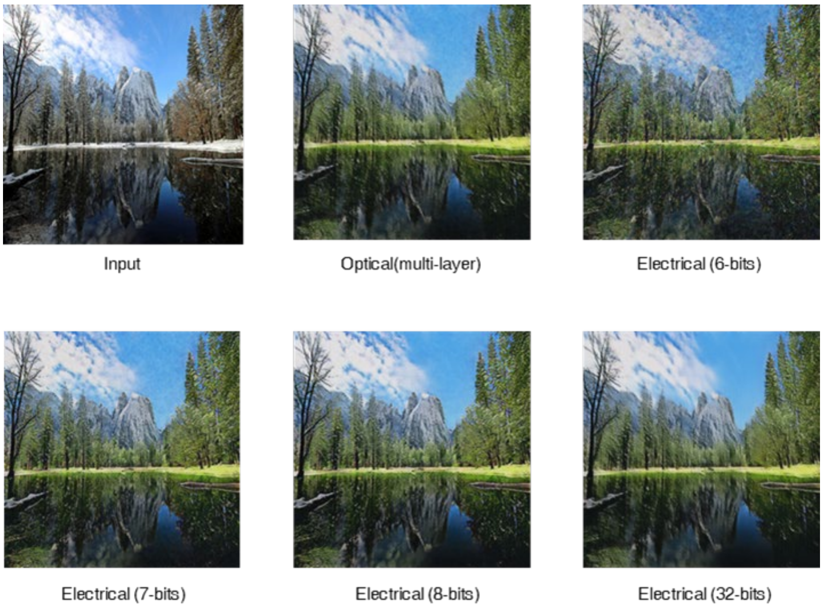

Fig. S27 Output results of seasonal transformation under different computing environments

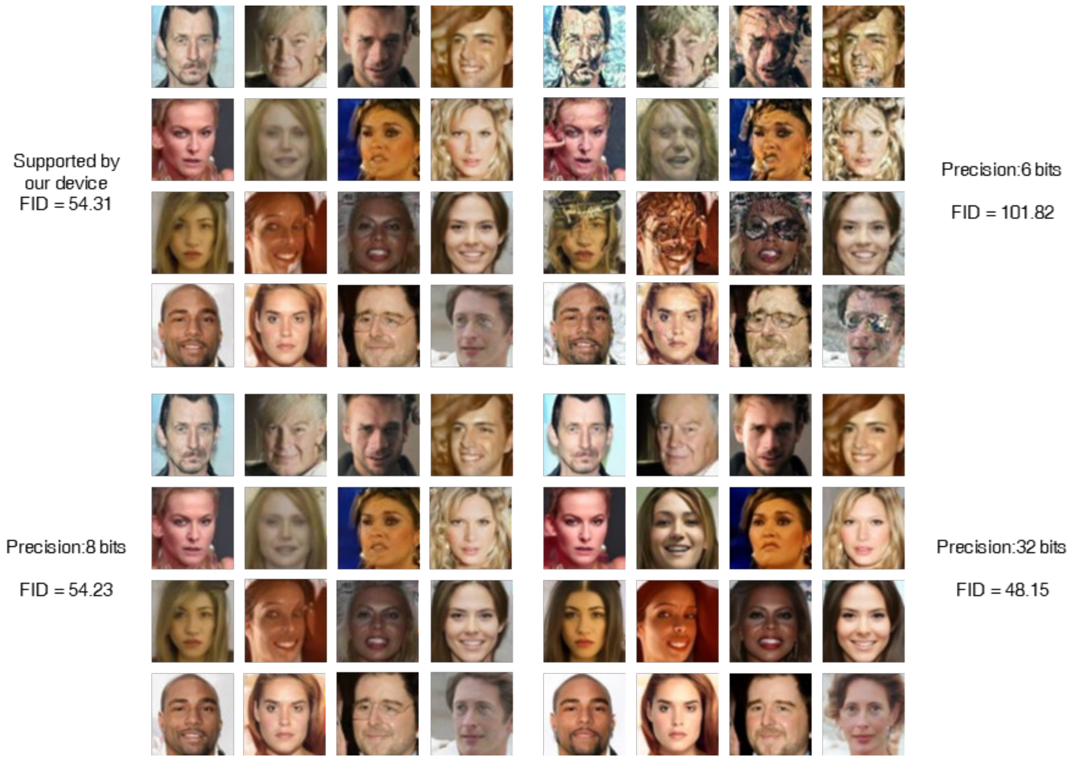

Fig. S28 The impact of computational precision on the output results in DDIM with the aid of QNCD

Table S4 List of instruments and components used in the measurement and characterization setups

**Equipment**	**Model**
Arbitrary Waveform Generator	Keysight M8194A
Oscilloscope	Keysight UXR0594BP
Optical Spectrum Analyzer	Yokogawa AQ6370D
Mach-Zehnder Modulator	Sumicem T.MXH 1.5-20PD-ADC-LV
Photodiode	Finisar XPDV4121R
Tunable laser	TSP-400-E0018
Erbium-Doped Fiber Amplifier	OVLINK EDFA-C-BA-GF-26-PM-B<br>OVLINK EYDFA-C-HP-BA-30-PM-B<br>Amonics AEDFA-23-B-FA
Wavelength Selective Switch	Finisar Waveshaper 4000S
Optical Band-Pass Filter	EXFO XTM-50-SCL-U
Variable Optical Attenuator	OVLINK SVOA-1000
Electrical Amplifier	AT-PA-1840-3330GN<br>SHF-S807C
intensity modulator	MXAN-LN-40
phase modulators	PM-DV5-40-PFA-PFA-LV
RF synthesizer	Agilent, 83630B
Broadband Isolation Balun	BAL-0026

Table S5 List of state-of-the-art communication devices on the SOI platform

Component	Loss	Reference
Programable Optical Filter	-2 dB	[4]
Directional coupler	-1.1 dB	[14]
Optical Filter	-1.1 dB	[15]

Table S6 Estimated energy consumption of the OPU

Component	Equation	numbers	Energy consumption
MZM	$P_{MRM} = V_{bias}I_{bias}$	8	5 mW
PD	$P_{PD} = RVP_r$	12	3.9 mw
Programable Optical Filter	From ref. 2	1	20 mw
**Total of computation**	$P_{Mzm} \cdot 8 + P_{PD} \cdot 12 + 20mW$	21	106.8 mw
Laser	$P_l = (P_{laser} + P_{TEC})/\eta$	1	137.7 mW
MRM	$P_{MZM} = V_{heater}I_{heater} + V_{bias}I_{bias}$	1	5.8 mw
PD	$P_{PD} = RVP_r$	1	3.9 mw
**Total of communication**	$P_L + P_{MRM} + P_{PD}$	3	147.4 mw
DAC	From ref. [16]	9	40mW
ADC	From ref. [17]	7	0.02mW
**Total of control plane**	$P_{DAC} \cdot 8 + P_{ADC} \cdot 6$	16	360.14mW

Reference

1. Zhu, H. H. *et al.* Space-efficient optical computing with an integrated chip diffractive neural network. *Nat Commun* **13**, 1044 (2022).
2. Shi, Y. *et al.* Nonlinear germanium-silicon photodiode for activation and monitoring in photonic neuromorphic networks. *Nat Commun* **13**, 6048 (2022).
3. Zhang, W. *et al.* Silicon microring synapses enable photonic deep learning beyond 9-bit precision. *Optica* **9**, 579 (2022).
4. Wang, M., Chen, X., Khan, U. & Bogaerts, W. Programmable wavelength filter with double ring loaded MZI. *Sci Rep* **12**, 1482 (2022).
5. Huang, R. *et al.* Low-Loss Silicon Photonic 16 × 16 Cyclic AWGR Based on SOI Platform. *IEEE Photonics Journal* **14**, 1–7 (2022).
6. Isola, P., Zhu, J.-Y., Zhou, T. & Efros, A. A. Image-To-Image Translation With Conditional Adversarial Networks. in *Isola_2017_CVPR* 1125–1134 (Isola_2017_CVPR, 2017).
7. Zhu, J.-Y., Park, T., Isola, P. & Efros, A. A. Unpaired Image-To-Image Translation Using Cycle-Consistent Adversarial Networks. in 2223–2232 (2017).
8. Dettmers, T. & Zettlemoyer, L. The case for 4-bit precision: k-bit Inference Scaling Laws. in *Proceedings of the 40th International Conference on Machine Learning* 7750–7774 (PMLR, 2023).
9. Kumar, T. *et al.* Scaling Laws for Precision. Preprint at https://doi.org/10.48550/arXiv.2411.04330 (2024).
10. Park, Y., Hyun, J., Cho, S., Sim, B. & Lee, J. W. Any-Precision LLM: Low-Cost Deployment of Multiple, Different-Sized LLMs. Preprint at

https://doi.org/10.48550/arXiv.2402.10517 (2024).

11. Egashira, K., Vero, M., Staab, R., He, J. & Vechev, M. Exploiting LLM Quantization. Preprint at https://doi.org/10.48550/arXiv.2405.18137 (2024).

12. Chu, H., Wu, W., Zang, C. & Yuan, K. QNCD: Quantization Noise Correction for Diffusion Models. in *Proceedings of the 32nd ACM International Conference on Multimedia* 10995–11003 (Association for Computing Machinery, New York, NY, USA, 2024). doi:10.1145/3664647.3681451.

13. Liu, Z., Luo, P., Wang, X. & Tang, X. Deep Learning Face Attributes in the Wild. in 3730–3738 (2015).

14. Cheben, P. *et al.* Refractive index engineering with subwavelength gratings for efficient microphotonic couplers and planar waveguide multiplexers. *Opt. Lett.* **35**, 2526 (2010).

15. Sun, C. *et al.* Tunable narrow-band single-channel add-drop integrated optical filter with ultrawide FSR. *PhotoniX* **3**, 12 (2022).

16. Lin, W.-T., Huang, H.-Y. & Kuo, T.-H. A 12-bit 40 nm DAC Achieving SFDR > 70 dB at 1.6 GS/s and IMD < –61dB at 2.8 GS/s With DEMDRZ Technique. *IEEE Journal of Solid-State Circuits* **49**, 708–717 (2014).

17. Tang, X. *et al.* Low-Power SAR ADC Design: Overview and Survey of State-of-the-Art Techniques. *IEEE Transactions on Circuits and Systems I: Regular Papers* **69**, 2249–2262 (2022).
